# Whole blood transcriptome profile at hospital admission discriminates between patients with ST-segment elevation and non-ST-segment elevation acute myocardial infarction

**DOI:** 10.1038/s41598-020-65527-7

**Published:** 2020-05-26

**Authors:** Mattia Chiesa, Luca Piacentini, Elisa Bono, Valentina Milazzo, Jeness Campodonico, Giancarlo Marenzi, Gualtiero I. Colombo

**Affiliations:** 1 Unit of Immunology and Functional Genomics, Centro Cardiologico Monzino IRCCS, Milano, Italy; 2 Unit of Intensive Cardiac Care, Centro Cardiologico Monzino IRCCS, Milano, Italy

**Keywords:** RNA sequencing, Transcriptomics, Biomarkers, Cardiology

## Abstract

Whether ST-segment (STEMI) and non-ST-segment elevation myocardial infarction (NSTEMI) should be regarded as distinct pathophysiological entities is a matter of debate. We tested the hypothesis that peripheral blood gene-expression profiles at presentation distinguish STEMI from NSTEMI. We performed a case-control study collecting whole-blood from 60 STEMI and 58 NSTEMI (defined according to the third universal definition of MI) consecutive patients on hospital admission. We used RNA-sequencing for the discovery phase, comparing 15 STEMI *vs*. 15 NSTEMI patients, matched for age, sex, and cardiovascular risk factors, and quantitative PCR in the remaining unmatched patients for validating top-significant genes. Gene-level differential expression analysis identified significant differences in the expression of 323 genes: 153 genes withstood correction for admission cardiac troponin I (cTnI), differentiating the two conditions independently of myocardial necrosis extent. Functional annotation analysis uncovered divergent modulation in leukocyte and platelet activation, cell migration, and mitochondrial respiratory processes. Linear regression analysis revealed gene expression patterns on admission predicting infarct size, as indexed by cTnI peak (R^2^ = 0.58–0.75). Our results unveil distinctive pathological traits for these two MI subtypes and provide insights into the early assessment of injury extent. This could translate into RNA-based disease-specific biomarkers for precision diagnosis and risk stratification.

## Introduction

Acute myocardial infarction (AMI) is a multifactorial disease that, despite considerable advances in prevention and treatment, is a leading cause of morbidity and mortality worldwide^1^. AMI is traditionally classified as ST-segment (STEMI) or non-ST-segment elevation myocardial infarction (NSTEMI), which present significant differences in clinical characteristics, prognosis and treatment options and timing^2^. Nonetheless, whether STEMI and NSTEMI are two different pathophysiological entities or nuances of a disease continuum is an object of controversy. On one hand, it is widely accepted that patients with STEMI and NSTEMI share similar risk factors, demographics, pathological substrate, complications, and tools for secondary prevention^1,3^. On the other, they display peculiar features^1^: STEMI patients present a transmural event with an occlusive coronary thrombus; conversely, patients with NSTEMI typically have a sub-endocardial occurrence with an incomplete or transient obstruction in the culprit coronary artery. Most patients with recurrence tend to repeat episodes of the same AMI type^4^. In addition, significant differences in the prevalence of infarct-related coronary artery^5,6^ and in fibrinolytic activity^7^ between STEMI and NSTEMI patients have been reported.

Genome-wide molecular profiling represents a promising tool for addressing such contrasting evidence. Specifically, peripheral blood gene expression profiling is an informative approach to investigate disease-specific states and identify biomarkers that may reflect genetic predisposition and/or disease activity^8^. Blood is an ideal surrogate tissue for AMI studies^9^ because it includes inflammatory cells that are critical elements in the atherothrombotic process and make contact with the diseased endovascular lumen and as such may serve as reporters. The search for transcriptional signatures on the whole blood, rather than on cell subpopulations, has compelling advantages^10^: cell fractionation is burdened with sample handling artefacts, results in some degree of cell activation, increases sample-to-sample variability, and limits the scope of the investigation to a few cell types.

Previous studies reported on gene expression patterns in peripheral blood that correlate with the extent of coronary artery disease (CAD) and may predict the likelihood of major adverse cardiovascular events^11–14^. Nevertheless, scarce information is available on AMI^15–18^, and no studies separate STEMI from NSTEMI. Furthermore, transcriptional profiling was performed using microarrays, which are limited in dynamic range and coverage, and most studies were done on isolated peripheral blood mononuclear cells (PBMC)^15,17,18^.

Here, we hypothesize that whole-blood transcriptome profiling may provide an in-depth insight into the underlying pathophysiological landscape differentiating STEMI from NSTEMI. To this end, we made use of RNA sequencing (RNA-Seq), which allows defining precise expression maps of known and unannotated genes and provides a ground-breaking tool for systematic investigation of transcriptional units relevant to disease conditions^19^. Furthermore, to test the concept that the circulating transcriptome holds disease-related information with clinical applicability, we sought for possible associations with a marker of AMI severity (cardiac troponin).

## Methods

An expanded Methods section is available in the Supplementary Information file.

### Study design

**Figure 1:**
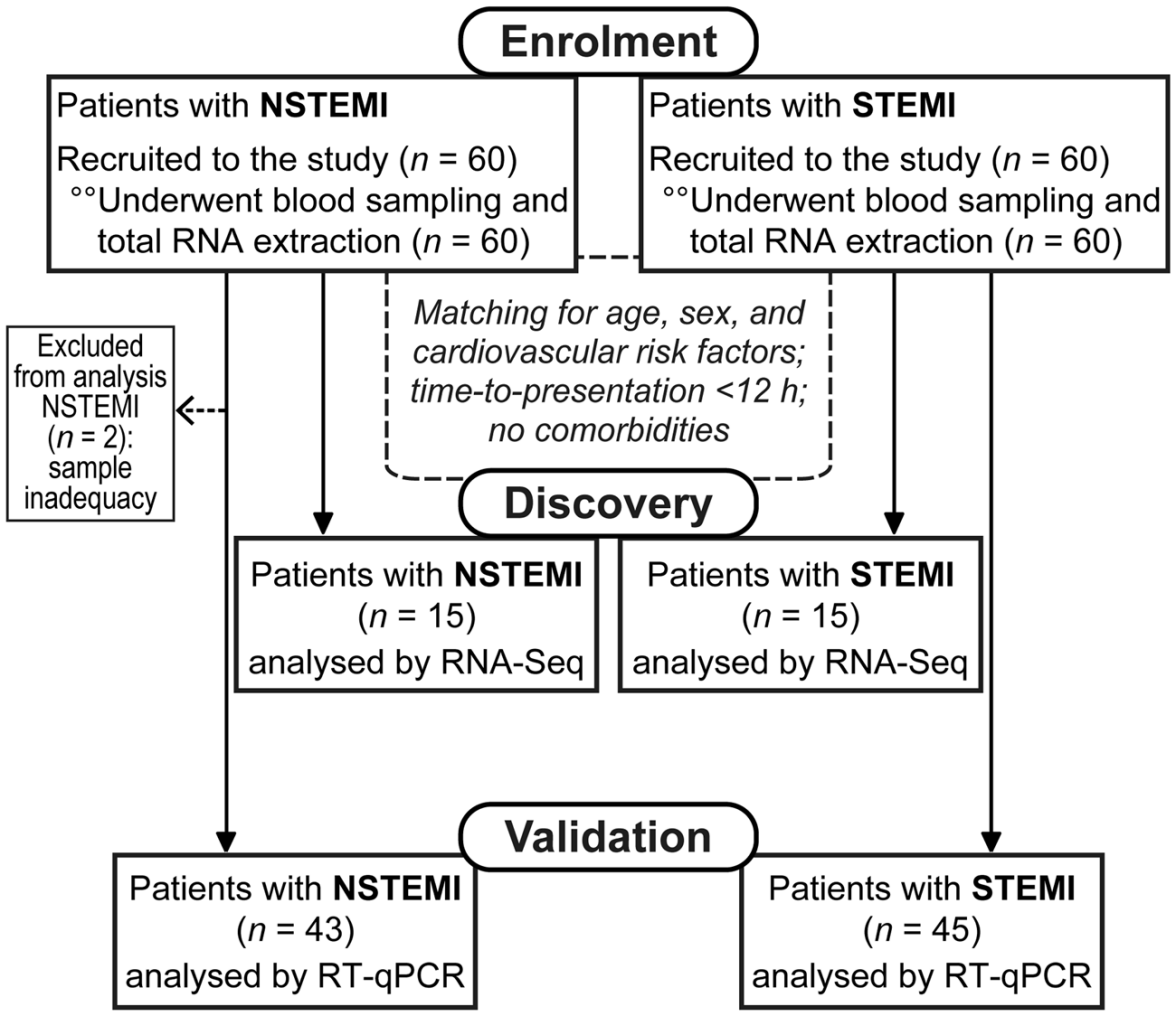
Flow diagram of the study.

One hundred and twenty consecutive patients admitted with STEMI or NSTEMI at our Centre between 2012 and 2015 were enrolled in this study (see flow diagram, Fig. 1).

STEMI and NSTEMI were defined according to the third universal definition of myocardial infarction^20^. In particular, STEMI was diagnosed as a typical chest pain lasting at least 30 min, with electrocardiographic ST-segment elevation of at least 0.2 mV in two or more contiguous leads, or left bundle branch block. NSTEMI was defined as recent (onset of symptoms ≤24 hours) characteristic chest pain with electrocardiographic ST-segment depression or T wave inversion and detection of a rise and/or fall of cardiac troponin I (cTnI) values. In all patients, the diagnosis of AMI was confirmed by coronary angiography. Exclusion criteria were: AMI with hemodynamic, electrical and mechanical complications at hospital presentation; relevant comorbidities, such as severe chronic kidney disease (estimated glomerular filtration rate <30 mL/min/1.73m^2^), chronic atrial fibrillation, prior stroke; other known cardiac diseases; systemic diseases, such as malignancy, infections or autoimmune diseases.

Peripheral blood samples were drawn from an antecubital vein into Tempus Blood RNA tubes (Applied Biosystems), containing RNA stabilizing reagents, on admission in the Intensive Cardiac Care Unit and before any medical intervention. To seek for specific differences between these two types of AMI and remove possible confounding factors, we performed an exploratory analysis by RNA-Seq selecting STEMI (*n* = 15) *vs*. NSTEMI (*n* = 15) patients matched for age, sex, and cardiovascular risk factors (CVRFs), with a time-to-presentation <12 hours and without relevant comorbidities, such as diabetes or chronic kidney disease (CKD). Following that, unmatched patients were used as a validation cohort for relevant biomarkers. Two NSTEMI patients were excluded from the analysis due to technical issues (sample degradation), whereas the remaining samples (STEMI *n* = 45 and NSTEMI *n* = 43) were analysed by reverse-transcription quantitative PCR (RT-qPCR).

The study protocol conformed to the principles of the Declaration of Helsinki. The “Ethics Committee of the IRCCS Istituto Europeo di Oncologia and Centro Cardiologico Monzino” approved the study protocol. All enrolled patients signed written informed consent. Participants also consented to share their de-identified information.

In the RNA-Seq discovery analysis, comparing 15 *vs*. 15 patients allowed achieving a statistical power of 99% to detect differences among the means ≥2.0 (effect size), with a biological coefficient of variation (BCV) = 0.2 (estimated from sequencing data obtained in preliminary experiments), a sequencing depth = 20 reads (corresponding to low expression levels), and a significance level α = 10^−4^.

The reporting of this study is in agreement with the STROBE statement (see Supplementary Information).

### RNA-sequencing

We assessed whole-blood gene expression profiles using RNase-free DNase-I-treated, globin-depleted, poly(A)+ RNA. Libraries were prepared and pooled together by a multiplex library RNA barcoding system and sequenced using the Sequencing by Oligonucleotide Ligation and Detection (SOLiD) approach (Applied Biosystems). Templates were paired-end sequenced [75 base pairs (bp) forward and 35 bp reverse].

We mapped reads to the human genome HG38/GRCh38.76 (*Ensembl* database) using TopHat v2.0.11 with Bowtie 1 to handle colour space reads^21^. To identify unannotated transcripts and quantified them along with well-annotated genes, we implemented the reference annotation based transcript (RABT) procedure and used the Cufflinks Suite v2.1.1^22^. See Supplementary Information for details.

### Validation by RT-qPCR

We performed first a technical and then a biological validation of the RNA-Seq data on selected genes by RT-qPCR in the study and in the validation cohorts, respectively. Primers and probes were chosen among predesigned and validated Applied Biosystems TaqMan Gene Expression Assays. Expression levels were normalized to the two most stable reference genes (*AP2A2* and *EIF3F*), identified using the NormFinder v0.953 Excel Add-In. We run qPCR with three replicates/sample for each assay on a ViiA 7 Real-time PCR System (Applied Biosystems). Data analysis was performed using the comparative Cq (ΔCq) method. See Supplementary Information for details.

### Differential gene expression analysis

Normalization procedures are crucial in RNA-Seq data analysis since they deeply affect the number and effect size of differentially expressed (DE) genes detected. Thus, we performed differential expression analysis controlling for “unwanted variation” (*e.g*., technical batch effects or other unknown confounding variables) using the between-sample normalization method (R package *RUVSeq*)^23^. A set of empirical negative control genes, supposed not to be influenced by the biological variables of interest (*i.e*., the AMI phenotype), was used to estimate factors of unwanted variations (*i.e*., *K* parameter of the *RUVg* method). The number of *k* factors was selected by comparing unadjusted with adjusted expression data by using diagnostic plots such as relative log expression plots, scatter plots of the first two principal components, and histogram plots of the distribution of the *P*-values for testing differential expression between STEMI and NSTEMI. A *k* = 8 factor of unwanted variation was chosen in our setting since it showed the best trade-off between data adjustment and the risk of data overcorrection.

We used the negative binomial generalized linear model (GLM) approach implemented in the edgeR package to perform differential expression analysis between STEMI and NSTEMI^24^. We deemed genes as significantly different at a false discovery rate (FDR)-adjusted *P*-value < 0.05. Analyses were performed both without and with correction for cTnI levels at presentation, assuming that transcriptional responses and expression levels are influenced both by disease-specific phenotypes and the entity of the cardiac damage after AMI.

For clustering analysis, we used the expression matrix of the log_2_-transformed normalized counts of the DE genes STEMI vs. NSTEMI samples. To draw the heatmap and the clustering dendrogram, we used an unsupervised method based on the dissimilarity matrix computed as Spearman rank correlation and the average linkage method implemented in the GENE-E software v3.0.215.

### Functional enrichment analysis on genome-wide expression profiles

To infer the biological functions associated with the AMI phenotypes, we took advantage of prior biological knowledge on genes grouped by Gene Ontology (GO) Biological Processes (BP) and used GO terms for Gene Set Enrichment Analysis (GSEA software v2.2)^25^. For GSEA we used the gene set collection repository of the Bader Lab (http://download.baderlab.org/EM_Genesets). The GSEA pre-ranked tool option was adopted and gene ranking metric was based on the likelihood ratio statistics of the differential expression analysis. Parameters used for analysis included 10000 permutations and limits to the gene sets (number of genes ranging from 8 to 500). To visually interpreting biological data, networks of the most significant GO-BP (at an FDR < 0.05) were drawn through the Enrichment Map software v3.0.0^26^, implemented as a plug-in in the Cytoscape v3.4.0 platform^27^.

A similar approach was used to perform a cell-type enrichment analysis. We created a custom gene-set collection integrating the 22 subsets of human hematopoietic cell types reported by Newman *et al*.^28^ with a platelet specific gene-set. The platelet gene-set was obtained merging the “Platelet activation, signalling and aggregation” (R-HSA-76002) and the “Platelet homeostasis” (R-HSA-418346) gene-sets from the Reactome database (http://www.reactome.org/). The final set of 80 platelet specific genes was drawn based on a log_2_(mean expression level)> 5.0 according to the dataset by Simon *et al*.^29^.

### Statistical analysis

Demographics categorical data are presented as counts and proportions, continuous data as the median and interquartile range (Q1–Q3). Gaussian distribution was tested using the D’Agostino-Pearson omnibus normality test. Categorical variables were compared by Fisher’s exact test. Given that many continuous variables did not pass the normality test, between-group comparisons were performed by the Mann-Whitney test. Analyses were done using GraphPad Prism v7.04 (GraphPad Software, La Jolla, CA). *P*-values < 0.05 were considered statistically significant.

To investigate the relationships between peripheral blood gene expression on hospital admission and infarct extent, we used linear regression models. As an estimate of infarct size, we used the peak cTnI level^30^. For each gene, we fitted two models with peak cTnI value (in log_2_ scale) as the response variable. In the first one, the gene expression levels are the unique predictors. In the second one, we added admission cTnI level as a covariate, assuming that it is a predictor of cTnI peak, in order to test whether expressed genes had independent effects. All models were fitted using the ‘lm’ function, implemented in R v3.4.0. The Benjamini-Hochberg procedure was used to control FDR. Multiple linear regression analysis was performed and models with an FDR < 0.05 were considered significant.

We assessed the correlation between RT-qPCR average normalized expression values (ΔCq) and RNA-Seq mean normalized counts (in log_2_ scale), by computing the Pearson’s correlation coefficient (*r*), the coefficient of determination (*R*^2^), and the significance *P*-value.

## Results

### Study population characteristics

Baseline demographic, clinical and laboratory features of the study population are listed in Table 1. Patients selected for the exploratory phase (*n* = 30; STEMI *n* = 15 *vs*. NSTEMI *n* = 15) had no history of previous AMI or stroke and no incident diabetes or CKD. Most subjects were males (73%) and overweight. STEMI and NSTEMI patients had no substantial differences in blood tests (except in neutrophil count, which was higher in STEMI), body mass index, major medications on admission (including aspirin and statins), time-to-presentation after symptom onset, and left ventricular ejection fraction (LVEF). In the remaining cohort used for the validation phase (*n* = 88; STEMI *n* = 45 *vs*. NSTEMI *n* = 43; 2 NSTEMI patients excluded for sample inadequacy), NSTEMI patients were on average older than STEMI and more frequently presenting hypertension, hypercholesterolemia and/or previous AMI. Moreover, NSTEMI subjects had a longer time-to-presentation and a slightly higher LVEF than STEMI and were more frequently on chronic aspirin, β-blockers, and statins. As expected, the mean cTnI peak was significantly higher in STEMI patients in both cohorts.

**Table 1:** Patient clinical characteristics.

Demographics	Study cohort			Validation cohort		
	NSTEMI (*n* = 15)	STEMI (*n* = 15)	*P*-value	NSTEMI (*n* = 43)	STEMI (*n* = 45)	*P*-value
Males	11 (73%)	11 (73%)	1.0	34 (79%)	31 (69%)	0.33
Age (years)	60 (56–75)	65 (54–73)	0.85	76 (64–79)	69 (57–74)	0.03
BMI (kg/m^2^)	27.7 (26.4–29)	28.7 (25–30.8)	0.77	27 (25.3–28.9)	28.2 (25.7–30.2)	0.49
Risk factors						
Current smokers	4 (26%)	7 (46%)	0.45	11 (26%)	11 (24%)	0.39
Hypertension	11 (73%)	10 (67%)	1.0	32 (74%)	22 (49%)	0.02
Hypercholesterolemia	4 (27%)	7 (47%)	0.45	26 (60%)	13 (29%)	0.005
Diabetes	0	0		10 (23%)	6 (13%)	0.28
Previous AMI	0	0		12 (28%)	4 (9%)	0.03
Previous PCI	0	0		15 (35%)	6 (13%)	0.02
Previous CABG	0	0		5 (12%)	2 (4%)	0.26
Laboratory tests						
Erythrocytes (10^6^/µL)	4.6 (4.4–5.1)	4.8 (4.7–5.2)	0.28	4.6 (4.2–4.8)	4.8 (4.2–5.0)	0.11
Haemoglobin (g/dL)	14.7 (13.9–15.3)	14.9 (13.9–15.4)	0.68	13.8 (12.6–14.6)	14.1 (13.2–15.6)	0.03
Leukocytes (10^3^/µL)	9.3 (6.0–10.5)	11.5 (9.4–13.2)	0.02	8.1 (7.0–9.8)	10.7 (8.3–13.1)	0.0002
Neutrophils (10^3^/µL)	4.6 (4.4–7.4)	7.5 (6.4–8.6)	0.02	5.7 (4.3–7.6)	7.6 (5.8–10.7)	0.0007
Lymphocytes (10^3^/µL)	2.0 (1.4–2.6)	2.3 (2.2–2.8)	0.23	1.6 (1.1–2.1)	1.8 (1.4–2.4)	0.09
Monocytes (10^3^/µL)	0.5 (0.4–0.8)	0.5 (0.4–0.8)	0.97	0.6 (0.4–0.7)	0.6 (0.5–0.9)	0.12
Platelets (10^3^/µL)	193.0 (169.5–203.5)	215.0 (164.5–286.0)	0.77	204 (173–230)	210 (187–259)	0.20
Total cholesterol (mg/dL)	195.0 (161.0–218.8)	207.0 (170.0–221.5)	0.53	171.0 (148.5–195.3)	183.5 (169.5–206.0)	0.07
LDL-c (mg/dL)	113.5 (92.3–150.5)	129.0 (105.3–149.3)	0.43	101.0(80.5–135.3)	117.5 (102.0–146.5)	0.03
HDL-c (mg/dL)	42.5 (35.3–45.8)	37.0 (31.0–44.0)	0.23	40.5 (33.5–46.5)	42.0 (37.5–48.0)	0.45
Triglycerides (mg/dL)	111.0 (83.8–120.5)	136.0 (90.0–164.5)	0.19	91.5 (63.3–119.8)	112.5 (84.3–136.5)	0.09
Glycaemia (mg/dL)	122.0 (110.0–132.5)	133.0 (122.0–150.5)	0.12	125.0 (100.5–160.5)	136.0 (120.0–164.0)	0.11
HbA1c (mmol/mol)	37.4 (36.0–39.8)	36.0 (34.9–38.1)	0.15	38.9 (35.0–46.0)	37.1 (34.7–44.7)	0.60
Creatinine (mg/dL)	1.1 (0.9–1.2)	0.9 (0.8–1.0)	0.14	1.0 (0.8–1.1)	0.9 (0.7–1.1)	0.08
eGFR (mL/min/1.73m^2^)	66.8 (55.8–93.7)	83.1 (65.7–100.9)	0.16	76.3 (58.3–98.8)	85.8 (71.1–99.5)	0.18
cTnI on admission (ng/mL)	0.6 (0.3–1.9)	2.1 (1.1–10.6)	0.08	1.0 (0.4–2.5)	1.1 (0.2–7.8)	0.71
cTnI, peak value (ng/mL)	3.4 (1.9–5.8)	43.4 (17.2–67.7)	<0.0001	4.0 (1.5–8.9)	44.9 (14.8–72.9)	<0.0001
hs-CRP	4.9 (2.5–7.3)	2.6 (1.1–4.2)	0.11	5.5 (1.7–8.7)	3.8 (1.7–15.2)	0.42
Admission medications						
Aspirin	4 (27%)	4 (27%)	1.0	28 (65%)	18 (40%)	0.02
Beta-Blockers	1 (7%)	2 (13%)	1.0	21 (49%)	9 (20%)	0.007
ACEI/ARB	7 (47%)	6 (40%)	1.0	22 (51%)	14 (31%)	0.08
Statins	2 (13%)	1 (7%)	1.0	18 (42%)	8 (18%)	0.02
Time-to-presentation (hours)	7.0 (5.3–9.0)	5.0 (3.0–6.3)	0.18	10.5 (4.0–24.0)	4.5 (2.0–15.0)	0.04
LVEF (%)	57.5 (50.5–63.8)	52.0 (47.5–58.2)	0.19	54.5 (48.0–61.3)	50.0 (40.0–54.8)	0.02
In-hospital procedures						
Primary PCI	0	15 (100%)		6 (14%)	45 (100%)	
Early (<24 hours) PCI	11 (74%)	0		31 (72%)	0	
Non-urgent PCI	2 (13%)	0		3 (7%)	0	
Elective CABG	2 (13%)	0		2 (5%)	0	
Medical Therapy	0	0		1 (2%)	0	

Categorical variables are presented as counts (n) and proportions (%); quantitative variables are expressed as the median and interquartile range (Q1-Q3). AMI, acute myocardial infarction; PCI, percutaneous coronary intervention; CABG, coronary artery bypass grafting; LDL-c, low-density lipoprotein cholesterol; HDL-c, high-density lipoprotein cholesterol; HbA1c, haemoglobin A1c; eGFR, estimated glomerular filtration rate, based on the Modification of Diet in Renal Disease equation; cTnI, cardiac troponin I; hs-CRP, high-sensitivity C-reactive protein; ACEI, angiotensin-converting enzyme inhibitor; ARB, angiotensin-II receptor blocker; LVEF, left ventricular ejection fraction.

### Sequencing data

A total of 81.6 ± 16.5 million reads per sample of the study cohort was collected. Most of them (50.2 ± 9.6 million reads per sample) mapped in annotated regions, while the remaining mapped to unannotated loci (see Supplementary Fig. S1A). We identified 26681 expressed genes, of which 17513 were known and annotated, while the remaining 9168 were unannotated genes found in intergenic regions (see Supplementary Fig. S1B). Among the latter, 154 showed a coding potential, while 9016 should be considered as non-coding RNAs (see Supplementary Fig. S1C).

### Differences in gene expression

**Figure 2:**
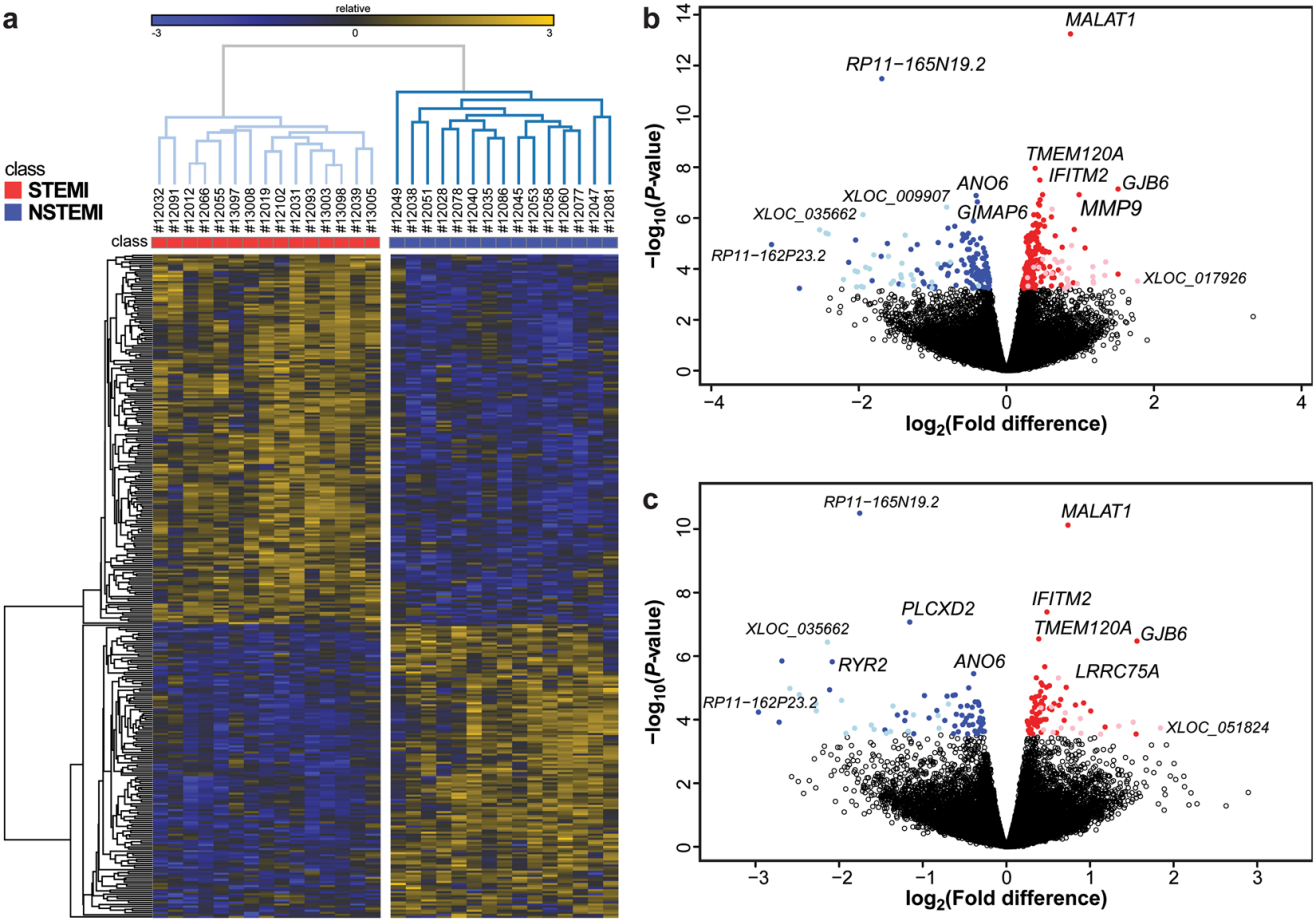
Differential gene expression in STEMI *vs*. NSTEMI matched patients. Statistical analysis was performed by negative binomial Generalized Linear Model, controlling for multiple testing using the false discovery rate (FDR) by the Benjamini-Hochberg procedure. (**a**) Heatmap depicting relative expression abundance of differentially expressed (DE) genes (FDR-adjusted *P* < 0.05) in STEMI (*n* = 15) *vs*. NSTEMI (*n* = 15) patients, matched for age, sex, and cardiovascular risk factors. Unsupervised average-linkage hierarchical clustering based on Spearman dissimilarity matrix allowed complete separation between STEMI and NSTEMI, suggesting that these 323 DE genes strongly associated with the specific AMI phenotype. Gene expression levels were expressed as log_2_ transformed normalized counts and displayed as gradient colours from higher (dark orange) to lower (dark blue). (**b**) Volcano plot depicting log_2_ mean fold-differences (STEMI *vs*. NSTEMI, *n* = 15 for both groups, x-axis) versus −log_10_ *P*-values (y-axis) of all genes, stemming from the differential analysis not corrected for the level of cardiac troponin I (cTnI) on admission. Significant DE genes are coloured: 151 annotated (red dots) and 29 unannotated (pink) genes were over-expressed in STEMI, whereas 113 annotated (blue dots) and 30 unannotated (light blue) genes in NSTEMI. (**c**) Volcano plot showing results of differential expression analysis in the same patient groups after correction for admission cTnI. Among the 153 DE genes standing the correction (FDR < 0.05), 64 annotated and 14 unannotated genes were over-expressed in STEMI and 57 annotated and 18 unannotated genes in NSTEMI. The average expression levels, the mean fold-differences, and the significance levels of all genes detected in STEMI (*n* = 15) *vs*. NSTEMI (*n* = 15) patients’ peripheral blood, for both the uncorrected and the cTnI-corrected models, are given in Supplementary Table S1.

To find robust differences between STEMI and NSTEMI transcriptomes, we performed differential gene expression analysis after correcting for unwanted confounding variables (see diagnostic plots in Supplementary Fig. S2). Using this approach, we detected 323 DE genes, with log_2_ fold-differences (STEMI *vs*. NSTEMI) ranging from −3.2 to 1.8 at an FDR < 0.05 (see Supplementary Table S1). Among them, 180 genes were expressed at higher levels in STEMI and 143 in NSTEMI patients (Fig. 2a,b). Significant genes ranged from very low to very high abundance (see Supplementary Fig. S3). Notably, 18% of DE genes were unannotated genes, of which 29 were over-expressed in STEMI and 30 in NSTEMI. Based on their sequence features, we predicted that 55 DE unannotated genes were putative long non-coding (longer than 200 nucleotides), 2 short non-coding (shorter than 200 nucleotides), and 2 protein-coding genes. DE genes were used to classify samples by unsupervised hierarchical clustering: Fig. 2a shows that the 323 DE genes clearly separate STEMI from NSTEMI patients.

To look for differences that are independent of the size of cardiac damage, we repeated differential expression analysis correcting for cTnI levels on admission and found 153 significant genes: 78 genes were expressed at higher levels in STEMI and 75 in NSTEMI (Fig. 2c and Supplementary Table S1).

### Functional inferences

**Figure 3:**
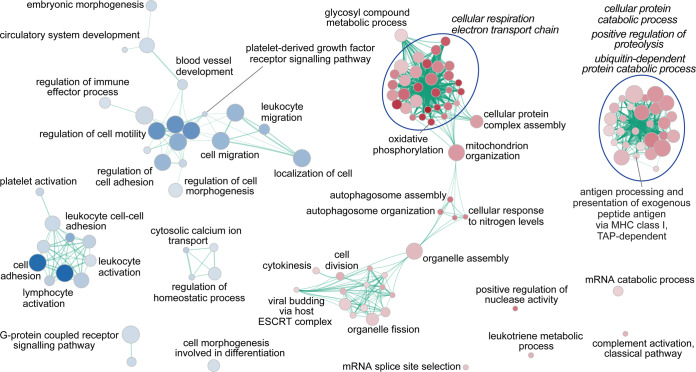
Enrichment map of gene-sets stemming from the analysis on the cTnI-corrected dataset. Functional enrichment investigation on genome-wide expression profiles was done by Gene Set Enrichment Analysis (GSEA), using as gene ranking metric the likelihood ratio statistics of the differential expression analysis performed by GLM, correcting for cTnI on admission, in STEMI (*n* = 15) *vs*. NSTEMI (*n* = 15) patients matched for age, sex, and cardiovascular risk factors. To visually interpreting GSEA results, a network of the most significant Gene Ontology biological processes (GO-BP; at an FDR-adjusted *P* < 0.05) was drawn. The node colour associates with STEMI (red) or NSTEMI (blue) phenotype; node gradient colour is proportional to node significance, from lower (light) to higher (dark); node size is proportional to the gene-set size. Edge thickness is proportional to the similarity between two gene-sets, for a cut-off of 0.25 of the combined Jaccard plus Overlap coefficient. An extended list of GO-BP gene-sets significant at a nominal *P*-value < 0.05, along with enrichment statistics, is given in Supplementary Table S2a.

Biological functions associated with the STEMI and NSTEMI phenotypes were inferred by GSEA on both uncorrected and cTnI-corrected statistic gene ranks, based on GO-BP (see Supplementary Table S2a). Using uncorrected data, we found that 97 biological processes were significantly associated with STEMI (FDR-adjusted *P* < 0.05) and 9 with NSTEMI. When correcting for cTnI on admission, 86 and 36 gene-sets were associated with STEMI and NSTEMI, respectively. To facilitate visualization and interpretation, enrichment networks were drawn for both GSEA uncorrected (see Supplementary Fig. S4) and corrected results (Fig. 3). The most significant and/or larger overview terms associated with STEMI were related to mitochondrial respiratory and electron transport chain, autophagosome assembly, and proteolysis, both in the uncorrected and in the corrected model. Conversely, NSTEMI was steadily associated with gene-sets involved in cell migration and adhesion and in G-protein coupled receptor signalling pathways, but only after correction for cTnI levels at presentation with blood vessel development and blood cell activation.

**Figure 4:**
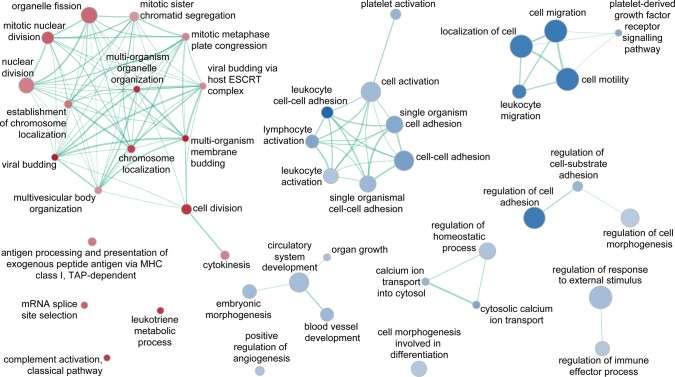
Enrichment map of gene-sets unique to the admission cTnI-corrected dataset. Functional enrichment analyses were done by GSEA, using as gene ranking metrics the likelihood ratio statistics of the differential expression analyses performed by GLM either correcting or not for cTnI on admission (in STEMI *vs*. NSTEMI patients, *n* = 15 for both groups, matched for age, sex, and cardiovascular risk factors). The map derived by subtracting the GSEA results obtained using the uncorrected gene ranks from those on the cTnI-corrected gene ranks. The colour scheme is as in Fig. 3. The complete lists of GO-BP gene-sets significant at a nominal *P*-value < 0.05, along with enrichment statistics, in either the uncorrected or the cTnI-corrected models, are given in Supplementary Table S2a.

A comparison between non-corrected and cTnI-corrected GSEA results (see Supplementary Fig. S5), filtering out gene-sets that were in common and retaining those specifically enriched in the cTnI-corrected dataset, allowed focusing on those GO-BP that most probably distinguish the two AMI phenotypes independently of cardiac damage extent. The resulting enrichment network consisted of 18 gene-sets associated with STEMI and 27 with NSTEMI (Fig. 4). Of interest, the STEMI phenotype was specifically linked with cell division processes, complement activation, and Major Histocompatibility Complex (MHC) class-I restricted antigen presentation, whereas NSTEMI with leukocyte adhesion, migration and activation, lymphocyte and platelet activation, and vessel development. Overlapping GO-BP (*i.e*., enriched both in the uncorrected and in the cTnI-corrected datasets; see Supplementary Fig. S6), included 68 gene-sets for STEMI (mitochondrial respiratory chain, protein and mRNA catabolic processes) and 9 for NSTEMI (cell motility and adhesion, G-protein coupled receptor signalling). The remaining 29 gene-sets resulting from the uncorrected dataset (see Supplementary Fig. S7) were overrepresented in STEMI patients only and comprised protein-targeting, regulation of protein ubiquitination, and redox processes.

### Cell-type enrichment

We inferred possible relationships between STEMI or NSTEMI phenotypes with specific cell-types through an enrichment analysis procedure. We performed this analysis both before and after correction for admission cTnI levels, to distinguish associations with STEMI or NSTEMI that were dependent or independent of cardiac damage extent at presentation (see Supplementary Table S2b). Our analysis showed that STEMI was associated with markers of neutrophils and plasma cells in response to cardiac injury (see Supplementary Fig. S8), but only neutrophils stood correction for cTnI (see Supplementary Fig. S9). This was consistent with the significantly higher number of neutrophils at blood count in STEMI patients (Table 1). Conversely, NSTEMI was consistently associated with immune cells, such as T and NK cells, whereas the association with antigen-presenting cells appeared related to cardiac damage response.

### Association with infarct size

**Figure 5:**
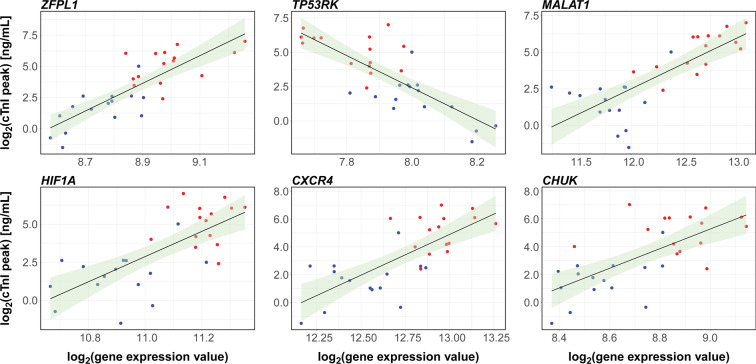
Genes predicting the cTnI peak. Linear regression was used to model the relationship between peak cTnI level (as the outcome variable) and peripheral blood gene expression on hospital admission (explanatory variable). Scatter plots and trendlines show six of the most significant genes that positively or negatively correlate with cTnI peak (see Table 2 for statistics). The full list of genes significantly predicting cTnI peak at an FDR-adjusted *P*-value < 0.05, along with the coefficients of determination *R*^2^, the regression *β* coefficients, and the exact *P*-values, in either the uncorrected or the cTnI-corrected models, is given in Supplementary Table S3. STEMI (*n* = 15) and NSTEMI (*n* = 15) samples are highlighted by red and blue dots, respectively. The 95% confidence interval of the trendline is depicted in light green.

To test whether the circulating transcriptome contains clinically relevant information, we assessed whether transcripts abundance at admission predicted the extent of the infarct size as indexed by cTnI peak, and found 551 models showing a significant association (FDR < 0.05) between gene expression level and peak cTnI value, being 314 genes positively and 237 negatively associated (see Supplementary Table S3). *R*^2^-values ranged between 0.32 and 0.69. When corrected for cTnI level on admission, 134 genes (81 with a positive and 53 with a negative regression *β* coefficient) were significant at an FDR < 0.05, with *R*^2^-values ranging from 0.58 to 0.75. Six of the top-ranked genes are shown in Fig. 5. Interestingly, only 36 out of the 134 genes resulting from the corrected analysis for admission cTnI, as well as 167 out of the 551 aforementioned genes not corrected for cTnI at presentation, showed significant differences in expression between STEMI and NSTEMI patients (see Supplementary Table S1).

### RT-qPCR validation

Changes in gene expression were first technically validated in the study cohort by RT-qPCR on 24 selected genes, including genes spanning from low to high-abundance expression levels, endogenous control genes, DE genes, and cTnI peak-associated genes. Normalized mean expression levels detected by qPCR and RNA-Seq showed a strong correlation, as ascertained by highly significant (*P* < 0.0001) Pearson’s coefficient (*r* = 0.91; see Supplementary Fig. S10).

**Figure 6:**
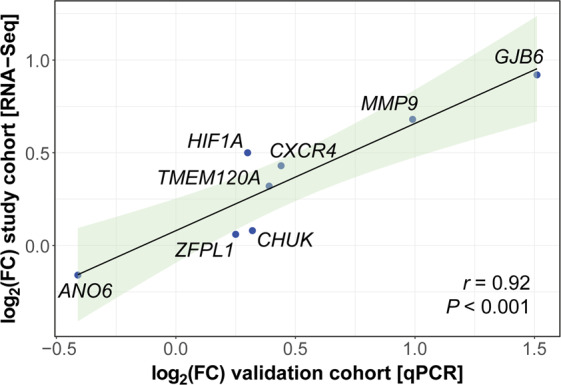
Validation of top-ranked genes on an independent cohort of consecutive patients. The expression level of 8 significant genes, chosen among the top DE genes and/or the top cTnI peak-associated genes, was evaluated in patients from the validation cohort using RT-qPCR single assays. Pearson’s correlation coefficient (*r*) was computed to assess the degree of association between the log_2_ mean fold-differences (log_2_FC) of these genes observed in the discovery group of matched patients (STEMI *n* = 15 *vs*. NSTEMI *n* = 15) and the log_2_FC found in the validation cohort (STEMI *n* = 45 *vs*. NSTEMI *n* = 43). Data are plotted as log_2_FC STEMI *vs*. NSTEMI in the study (y-axis) versus the validation patient cohort (x-axis). The 95% confidence interval of the trendline is depicted in light green.

We then selected 8 genes for RT-qPCR validation in the independent cohort of remaining, unmatched enrolled patients: 4 genes were chosen among the top 20 DE genes (*TMEM120A*, *GJB6*, *MMP9*, and *ANO6*) and 4 were among the top 20 genes associated with the cTnI peak (*ZFPL1*, *HIF1A*, *CXCR4*, and *CHUK*). *TMEM120A*, *ANO6*, *CXCR4*, and *HIF1A* were both DE and cTnI peak-associated genes. RT-qPCR results in the validation cohort largely corroborated both the significant differences in gene expression and the associations between the abundance of specific transcripts at admission and cTnI peak values observed in matched STEMI *vs*. NSTEMI patients by RNA-Seq. The mean fold-differences (significant or not) for the 8 genes were similar in the study and validation cohorts (Fig. 6). Almost all DE genes evaluated were significantly and consistently different in the validation cohort, both in the non-corrected and in the cTnI-corrected analysis (Table 2). *ANO6*, *CXCR4*, and *HIF1A* were significant also after adjustment for baseline variables showing an imbalance between STEMI and NSTEMI groups (age, hypercholesterolemia, hypertension, aspirin and statin use, time-to-presentation, admission cTnI). Similarly, genes associated with the cTnI peak in the study group showed significant associations also in the validation cohort, in both the non-corrected analysis, the model adjusted for cTnI level on admission, and the model fully adjusted for the abovementioned baseline covariates (Table 2).

**Table 2:** Validation of RNA-Seq data by RT-qPCR in an independent cohort.

Gene	Study cohort (*RNA-Seq*) (STEMI *n* = 15 *vs*. NSTEMI *n* = 15)						Validation Cohort (*RT-qPCR*) (STEMI *n* = 45 *vs*. NSTEMI *n* = 43)								
	*Non-corrected model*			*cTnI-corrected model*			*Non-corrected model*			*cTnI-corrected model*			Full model*		
**Differentially expressed genes**															
	**log**_**2**_**FC**		***P*-value**	**log**_**2**_**FC**		***P*-value**	**log**_**2**_**FC**		***P*-value**	**log**_**2**_**FC**		***P*-value**	**log**_**2**_**FC**		***P*-value**
*TMEM120A*	0.39		1.1 × 10^−8^	0.39		2.9 × 10^−7^	0.32		0.05	0.27		0.06	0.16		0.33
*GJB6*	1.51		7.2 × 10^−8^	1.56		3.4 × 10^−7^	0.92		0.01	0.84		0.009	0.61		0.09
*MMP9*	0.99		1.2 × 10^−7^	0.83		3.5 × 10^−5^	0.68		0.02	0.61		0.03	0.32		0.31
*ANO6*	−0.41		1.3 × 10^−7^	−0.39		3.6 × 10^−6^	−0.16		0.05	−0.18		0.036	−0.19		0.047
*CXCR4*	0.44		2.7 × 10^−7^	0.42		7.0 × 10^−6^	0.43		0.001	0.42		0.0004	0.34		0.008
*HIF1A*	0.30		1.7 × 10^−5^	0.31		5.6 × 10^−5^	0.50		0.003	0.48		0.002	0.42		0.02
**Genes associated with cTnI peak**															
	**R**^**2**^	***β***	***P*-value**	**R**^**2**^	***β***	***P*-value**	**R**^**2**^	***β***	***P*-value**	**R**^**2**^	***β***	***P*-value**	**R**^**2**^	***β***	***P*-value**
*ZFPL1*	0.69	11.34	1.5 × 10^−8^	0.75	9.90	2.2 × 10^−7^	0.06	2.02	0.05	0.10	1.73	0.01	0.25	1.30	0.003
*HIF1A*	0.54	8.63	3.4 × 10^−6^	0.71	7.64	1.3 × 10^−6^	0.15	1.37	0.0002	0.24	1.45	<0.0001	0.33	1.17	0.0001
*CXCR4*	0.54	5.90	3.4 × 10^−6^	0.67	5.05	9.1 × 10^−6^	0.20	2.00	<0.0001	0.35	2.49	<0.0001	0.41	2.12	<0.0001
*CHUK*	0.54	7.60	4.0 × 10^−6^	0.70	6.69	2.0 × 10^−6^	0.05	1.21	0.04	0.09	0.86	0.01	0.24	0.65	0.004
*TMEM120A*	0.49	7.20	1.6 × 10^−5^	0.63	6.13	3.6 × 10^−5^	0.12	1.36	0.0009	0.17	1.20	0.0005	0.29	0.97	0.0007
*ANO6*	0.49	−6.41	1.6 × 10^−5^	0.61	−5.34	8.9 × 10^−5^	0.03	−1.06	0.13	0.12	−1.47	0.004	0.26	−1.14	0.002

cTnI, cardiac troponin I; log_2_FC, fold-change in logarithmic scale, *i.e*., the difference between STEMI *vs*. NSTEMI. *In the full model comparison was adjusted for age, admission cTnI, hypercholesterolemia, hypertension, time-to-presentation, admission medications (aspirin and statins).

## Discussion

Comprehensive transcriptome profiling has been used to distinguish disease-specific mechanisms, which may provide diagnostic and prognostic value^8,9^. Herein, we compared whole-blood transcriptome profiles of STEMI and NSTEMI patients and proved the sensitivity of blood-based gene expression analysis by RNA-Seq in differentiating the two conditions. To focus on the most specific differences between the AMI types and reduce the effects of confounding factors, we compared STEMI and NSTEMI patients matched for age, sex, CVRFs and with no comorbidities. We then validated key observations on an independent cohort of unmatched patients. The primary finding was the identification of annotated and unannotated genes discriminating STEMI from NSTEMI: RNA-Seq unveiled new molecular players that could be useful for an in-depth understanding of the pathophysiological differences between STEMI and NSTEMI. Functional enrichment analysis showed that distinct, specific pathways and cell subpopulations were associated with the AMI type. Remarkably, correction for cTnI level on admission allowed distinguishing genes and regulated pathways primarily related to the AMI phenotype *per se* and not affected by the extent of cardiac damage. Indeed, our study shows a large number of significantly DE gene-sets that make up a complex scenario underlying the two phenotypes, which cannot be simplistically attributed to the extent of the myocardial damage but suggests that STEMI and NSTEMI are two distinct pathophysiological entities at the molecular level. Finally, we identified gene expression patterns on admission that predict peak cTnI elevation, *i.e*. the extent of myocardial necrosis. These findings indicate that blood-based gene expression profiling at the initial presentation is a sensitive, non-invasive tool that reveals transcriptional patterns, which anticipate the extent of myocardial injury in patients affected by acute ischemic heart disease. Overall, results confirmed our prior hypothesis and provided evidence that STEMI and NSTEMI have a distinct “molecular architecture”.

This study has several strengths worth mentioning. To our knowledge, this is the first study searching for AMI-subtype specific transcriptional differences in the whole blood by RNA-Seq. The general idea is searching for expression signatures that may have pathophysiological specificity and are not related to cardiac leakages, such as cTnI, which by nature is not specific for AMI. Blood was collected at patient admission, before any intervention, and without cell fractionation: this increases the reliability and robustness of the emerging biomarkers and makes more feasible a future clinical exploitation. On the other hand, clear limitations are the small sample size and selection criteria of the discovery cohort: while focusing on matched patients affected by AMI only (25% of our cases) increased the specificity and sensitivity of differential gene expression profiling, these constraints may reduce the generalisability of our results. However, we validated eight top DE and/or cTnI peak-associated genes on an independent cohort of consecutive, unmatched patients and, overall, we have confirmed our findings even when adjusting for relevant confounders.

Few previous studies meant to detect relevant expression changes in peripheral blood of AMI patients, using whole-genome microarray expression profiling. Circulating cell transcriptome was shown to reflect inflammatory and immune response to ischemic myocardial injury in first-time AMI patients within 48-hours post-MI, in comparison with normal controls, and modulation in epithelial-to-mesenchymal transition pathway or cholesterol transport were associated with disease severity and/or clinical outcome^17^. Alterations in PBMC gene expression patterns related to lipid/glucose metabolism, platelet function, and atherosclerotic plaque stability were observed in STEMI patients, on the 1^st^-day post-MI, when compared to stable CAD controls^15^. Upregulation of inflammatory genes and downregulation of genes involved in T-lymphocyte signalling were detected in peripheral blood samples of AMI patients collected immediately prior to angiography, in comparison with no-AMI subject with or without CAD, and a subset of these transcripts was associated with a significant risk of cardiovascular death^16^. Further, the extent of late microvascular obstruction, a cardiac magnetic resonance (CMR) surrogate marker of prognosis, was shown to correlate with upregulation of genes involved in inflammatory response, phagocyte mobilization, fatty acid utilization, and vascular dysfunction and downregulation of genes related to T-lymphocyte differentiation and activation in PBMC collected within 1 day from reperfusion in STEMI patients undergoing primary angioplasty^18^. Consistently, AMI was shown to activate inflammatory and proliferative pathways in circulating monocytes, prior to their infiltration of injured myocardium^31^. To this body of evidence, our study adds the notion that blood-based signatures of divergent modulation of inflammatory, immune-response, angiogenic, and mitochondrial dynamics networks characterize different types of AMI. NSTEMI and STEMI are considered a continuum of disease and a spectrum of clinical presentations following atherosclerotic plaque rupture and partial or complete thrombosis of the infarct-related artery^1^. Conversely, our findings showed that STEMI- and NSTEMI-specific expression patterns are distinguishable in peripheral blood, suggesting different pathophysiological traits.

Our data indicate a number of potential divergent molecular mechanisms. Three long non-coding RNAs (lncRNAs), *MALAT1*, *ZFAS1*, and *MIR17HG*, were significantly overexpressed in STEMI patients and, notably, *MALAT1* was also one of the best predictors of cTnI peak. LncRNAs are key regulators of tissue homeostasis and are involved in cardiac development, hypertrophy and remodelling, heart failure, and AMI^32^. MALAT1 regulates vessel growth and function and its expression may be influenced by hypoxia. Accordingly, we found that STEMI patients expressed higher levels of the hypoxia-inducible gene *HIF1A* than NSTEMI. It has been reported that AMI patients express higher levels of *MALAT1* compared with healthy controls, but slightly lower levels in STEMI than in NSTEMI^33^. The discrepancy with our findings may be due to the different study design (those authors collected blood samples at the time of reperfusion, *via* an arterial catheter) or the specific transcript detected (*via* RT-qPCR)^33^. *ZFAS1* was shown to be upregulated in the infarcted and border zones in a mouse model of AMI, and knockdown of *ZAFS1* protected cardiomyocytes from hypoxic injury^34^. Overexpression of *ZFAS1* detected in STEMI subjects may parallel increased myocardial hypoxia due to persistent total coronary occlusion. *MIR17HG* is the host gene for the MIR17–92 cluster, a group of microRNAs involved in cell survival, proliferation, differentiation, and angiogenesis: indeed, these were all pathways that differentiate STEMI from NSTEMI. Remarkably, we also found 29 DE, unannotated, predicted lncRNAs. In comparison with protein-coding mRNAs, lncRNAs show greater tissue/cell specificity^35^ and, being emerging regulators of cardiovascular functions, promise to improve phenotype discrimination and diagnostic and prognostic assessment.

The NSTEMI phenotype was associated with processes such as “blood vessel development”, “positive regulation of angiogenesis”, “cell migration”, and “regulation of cell adhesion”, which suggests that long-lasting history of CAD and/or transient ischemia may have triggered early mechanisms to help to restore damaged vessels and limit cardiomyocyte loss. Conversely, consistent with the prompt mobilization of angiogenic bone marrow cells and monocytes reported in AMI^36^, the chemokine *CXCR4* was significantly more expressed in STEMI than in NSTEMI patients. Patients with recent NSTEMI were shown to have a lower microvascular density in non-ischemic myocardium than patients with a similar extent of CAD without previous AMI^37^. Our data are in line with the proposal that acute coronary syndrome presentation depends not only on the presence of vulnerable plaque but also on the microcirculation dysfunction of a vulnerable myocardium^38^.

A group of interconnected gene-sets (“cell-cell adhesion”, “leukocyte/lymphocyte activation”, “platelet activation”) were over-represented in NSTEMI *vs*. STEMI patients. Of note, *ANO6*, which encodes for a key component of the calcium-dependent exposure of phosphatidylserine on the cell surface, is essential to trigger the clotting system^39^. The interplay between the haemostatic and inflammatory systems has a key role in atherosclerosis progression^40^: platelets can adhere to and be activated on stimulated endothelial cells promoting the recruitment of blood-borne leukocytes to the vessel wall, and formation of heterotypic platelets-leukocytes aggregates occurs in the blood prior to contact with endothelial cells. These enriched processes at presentation may reflect pathogenic mechanisms that differ between NSTEMI and STEMI.

A unique feature of the STEMI phenotype was the association with “antigen processing and presentation of exogenous peptide antigen *via* MHC class I, TAP-dependent” and proteolytic machinery gene-sets, suggesting immune tolerance breaking mechanisms occurring during a sterile injury such as AMI. Tissue necrosis prompts dendritic cells (DCs) to activate cardiac-specific autoreactive T-cells making the heart vulnerable to an autoimmune response^41^, as it was observed for cardiac myosin^42^. Recruitment of circulating DC precursors into the infarcted myocardium is paralleled by reduced numbers of circulating DCs in AMI, with a more pronounced reduction in STEMI than in NSTEMI patients^43^. Consistently, our analysis inferred that circulating DCs were associated with NSTEMI, whereas lower circulating DCs in STEMI might reflect recruitment into the infarcted myocardium, which in turn increases local inflammation and autoantigen presentation.

Mitochondrial dynamics and cellular respiration pathways appeared substantially altered in STEMI compared to NSTEMI patients. Dysregulated reactive oxygen species production in response to stress induces mitochondrial dysfunction and cell death, including apoptosis triggered by cytochrome c release. Cardiomyocytes have intrinsic quality control mechanisms to maintain energy balance and overall health of mitochondria, including fission, fusion, and autophagy^44^. A number of interconnected gene-sets related to mitochondrion organization and redox processes were clearly associated with the STEMI phenotype, mainly in response to cardiac damage, suggesting that both energy balance and autophagy mechanisms are activated and play a role during a massive ischemic event.

A highly promising finding is the association between blood cell gene expression on hospital admission and a recognized index of disease severity. Risk stratification of AMI patients at initial presentation is essential for optimal management. Peak troponins level is greatly related to infarct size^45^, but peak elevation usually occurs hours after AMI, and troponins on admission poorly predict the extent of cardiac injury^46^. We provide evidence that expression levels of specific genes in the peripheral blood on admission had a significant relationship with cTnI peak, independent of cTnI level at presentation: this should be regarded as a proof of concept that they might be early surrogate predictors of myocardial necrosis and infarct size. Thus, circulating transcriptional signatures may be valuable tools for early prognosis and risk assessment in AMI. Indeed, troponins have emerged as powerful predictors of prognosis^47,48^, and infarct size detected by CMR predict a wide array of adverse cardiovascular events^49^. Our findings suggest that RNA-based biomarkers may add valuable information for very early assessment of the risk for adverse cardiovascular outcomes, beyond that provided by troponins.

Refined prediction models may translate molecular findings into clinical applications, extending physician’s tools for appropriate decision-making and treatment plan. Indeed, CK-MB and cTnI have been proposed for the assessment of the cardioprotective effect of conditioning therapies, because of their availability and their known correlation with infarct size^50^. Early and accurate infarct size estimations by RNA-based disease-specific biomarkers could serve for the timely choice of appropriate cardioprotective therapies on ischemia and/or reperfusion-induced lesions.

### Study limitations

In interpreting our data, some limitations should be acknowledged. First, we considered peak cTnI concentration as a marker of infarct size. Despite troponins have been validated against histology and have demonstrated to be closely correlated with infarct size and prognosis in clinical practice^51,52^, our data should be confirmed by more accurate imaging markers of infarct size estimation, such as single-photon emission computed tomography (SPECT) myocardial perfusion imaging or CMR. Second, patients with major comorbidities and complications were excluded in our pilot study. Therefore, the applicability of our findings to these patients needs further investigation. Third, the translation of our results to daily clinical practice remains to be clarified and will require further studies.

## Conclusions

In conclusion, we showed that capturing global genomic responses through changes in mRNA expression in the blood unveiled molecular signatures and unappreciated distinct pathways for STEMI and NSTEMI and revealed early predictors of infarct size. Our analysis indicates specific pathological traits of these two forms of AMI that could provide a framework for the development of novel blood-based, disease-specific biomarkers for precision diagnosis, early risk stratification, and therapeutic decision-making.

## Supplementary information

Supplemental Material.

Supplemental Material 2.

Supplemental Material 3.

Supplemental Material 4.

## Data Availability

The RNA-Seq dataset generated and analysed during the current study, *i.e*., anonymized raw and processed, MIAME-complaint RNA-Seq data, is available in the NCBI GEO repository under the accession number GSE103182 (http://www.ncbi.nlm.nih.gov/geo/query/acc.cgi?acc=GSE103182). All other data generated or analysed during this study are included in this published article and its Supplementary Information files.
