# Supplementary material for: Whole blood transcriptome profile at hospital admission discriminates between patients with ST-segment elevation and non-ST-segment elevation acute myocardial infarction
Source: Sci Rep. 2020 May 26;10:8731. doi: 10.1038/s41598-020-65527-7 (PMC7250845; doi:10.1038/s41598-020-65527-7)
Supplement: Supplementary file 1 — Supplemental Material. [file 41598_2020_65527_MOESM1_ESM.pdf]

SUPPLEMENTARY INFORMATION

Whole blood transcriptome profile at hospital admission discriminates between patients with ST-segment elevation and non-ST-segment elevation acute myocardial infarction

Mattia Chiesa[1]†, Luca Piacentini[1]†, Elisa Bono[1], Valentina Milazzo[2], Jeness Campodonico[2], Giancarlo Marenzi[2], Gualtiero I. Colombo[1]*

[1]Unit of Immunology and Functional Genomics and
[2]Unit of Intensive Cardiac Care, Centro Cardiologico Monzino IRCCS, Milano, Italy.

†*MC and LP contributed equally to this work.*

****Corresponding author**:*
Dr Gualtiero I. Colombo, MD, PhD
Head, Lab. of Immunology and Functional Genomics
Centro Cardiologico Monzino IRCCS
Via Carlo Parea, 4 – 20138 Milano – Italy
T +39 02 5800.2464 - F +39 02 5800.2750
E-mail gualtiero.colombo@cardiologicomonzino.it

SUPPLEMENTARY METHODS

Blood sample collection

Peripheral blood samples were drawn from an antecubital vein on admission in the Intensive Cardiac Care Unit into Tempus Blood RNA tubes (Applied Biosystems, Foster City, CA) before any medical intervention. Tempus tubes contain RNA stabilizing reagents that lyse whole blood cells and prevent RNA degradation and/or changes in transcript relative composition. To obtain complete lysis, tubes were vortexed for ≥ 10 seconds and, then, stored at –80°C.

RNA isolation, library preparation, and sequencing

We isolated total RNA using the Tempus Spin RNA Isolation Kit (Applied Biosystems) and performed RNase-free DNase-I treatment to eliminate genomic contamination, following the manufacturer's instructions. We assessed RNA quantification and purity by micro-volume spectrophotometry on an Infinite M200 PRO multimode microplate reader (Tecan, Männedorf, Switzerland). We then checked RNA quality and integrity by microfluidics electrophoresis using the RNA 6000 Nano Assay Kit on a 2100 Bioanalyzer system (Agilent Technologies, Santa Clara, CA).

Five µg of total RNA were precipitated, α- and β-globin mRNAs were depleted with GLOBINclear Whole Blood Globin Reduction kit (Applied Biosystems), and poly(A)+ RNAs were enriched following MicroPoly(A) Purist kit protocol (Applied Biosystems). Libraries were prepared and pooled together using multiplex library RNA Barcoding reagents and Total RNA-Seq kits for the Sequencing by Oligonucleotide Ligation and Detection (SOLiD) System (Applied Biosystems). Complementary DNA (cDNA) amplification reaction was conducted with a 20-µL template in 100-µl end-volume reaction and with 16 PCR cycles. Clonal amplification of library templates was performed on SOLiD p1 DNA beads by emulsion PCR (ePCR) using a 0.5 pM library template and E120 EZbeads scale. Three different sample libraries were seeded in each lane of a SOLiD flow chip (400 million p2-EZBeads) and templates were paired-end sequenced [75 base pairs (bp) forward and 35 bp reverse] on a SOLiD 5500xl System (Applied Biosystems).

Data pre-processing

Using XSQTools (Applied Biosystems) with default parameters, we removed reads with low-quality base values and generated .csfasta and .QV.qual files for each sample. Using TopHat v2.0.11 with Bowtie 1[1,2] to handle colour

space reads, we mapped all reads to the human genome version HG38/GRCh38.76 downloaded from the *Ensembl* database[3]. We excluded 'haplotypes' and 'patches' sequences from the reference, in order to focus on the primary assembly and to avoid under-estimation of gene expression. Then, we implemented the reference annotation based transcript (RABT) procedure, using Cufflinks suite v2.1.1[4,5], to create a new assembly for our downstream analysis, integrating information about known genes with those reads mapped in intergenic or intronic regions. Using the latter annotation, we identified unannotated transcripts and quantified them along with well-annotated genes. We estimated genes and transcripts expression levels by Cuffquant (using default parameters, apart from -multi-read-correct -frag-bias-correct 'reference.fasta', and -max-bundle-frags 100,000,000) and then Cuffnorm (with default parameters, apart from -total-hits-norm). For each feature, we computed both reads counts and fragments per kilobase of transcript per million fragments mapped (FPKM) values. Genes with an FPKM value ≥ 0.046 in at least 60% of samples were considered expressed. This threshold was obtained by correlating RNA-Seq data with those from a qPCR gene expression array (TaqMan Array Human Inflammation, Applied Biosystems) in preliminary experiments (not shown).

Validation of RNA-Seq data by reverse transcription-quantitative PCR (RT-qPCR)

We performed both a technical and biological validation of the RNA-Seq data on selected genes by RT-qPCR, in the study and the validation cohorts, respectively. cDNA for single target gene expression analysis was synthesized from 2 µg of total RNA for each sample using the High Capacity cDNA Reverse Transcription Kit (Applied Biosystems). Primers and probes were chosen among predesigned and validated Applied Biosystems TaqMan Gene Expression Assays. To identify the most appropriate endogenous control genes among four candidates (*AP2A2*, *EIF3F*, *HPRT1*, and *GAPDH*), we performed an analysis of gene expression stability using the NormFinder v0.953 Excel Add-In[6]. Expression levels were normalized to the two most stable reference genes (*AP2A2* and *EIF3F*). Reagents, including 2× TaqMan Gene Expression Master Mix, and cDNA samples (20 ng/well) were handled with a MicroLab STAR automated liquid handling workstation (Hamilton Robotics, Bonaduz, Switzerland) to minimize dispensing errors. We run qPCR with three replicates/sample for each assay in a 384-well format plate on a ViiA 7 Real-time PCR System (Applied Biosystems). The experimental threshold and baseline were imputed by algorithms implemented in the ViiA 7 software v1.2. Data analysis was performed with the Expression Suite software v1.1 (Applied Biosystems), using the comparative Cq (ΔCq) method.

STROBE Statement—Checklist for observational case-control studies

	Item No	Recommendation	Page No
**Title and abstract**	1	(*a*) Indicate the study's design with a commonly used term in the title or the abstract	2
		(*b*) Provide in the abstract an informative and balanced summary of what was done and what was found	2
**Introduction**			
Background/ rationale	2	Explain the scientific background and rationale for the investigation being reported	3
Objectives	3	State specific objectives, including any prespecified hypotheses	3-4
**Methods**			
Study design	4	Present key elements of study design early in the paper	4
Setting	5	Describe the setting, locations, and relevant dates, including periods of recruitment, exposure, follow-up, and data collection	4
Participants	6	(*a*) Give the eligibility criteria and the sources and methods of case ascertainment and control selection. Give the rationale for the choice of cases and controls	4
		(*b*) For matched studies, give matching criteria and the number of controls per case	4<br>Tab.1
Variables	7	Clearly define all outcomes, exposures, predictors, potential confounders, and effect modifiers. Give diagnostic criteria, if applicable	4-6
Data sources/ measurement	8	For each variable of interest, give sources of data and details of methods of assessment (measurement). Describe comparability of assessment methods if there is more than one group	5-6<br>*Suppl.*<br>2-3
Bias	9	Describe any efforts to address potential sources of bias	5
Study size	10	Explain how the study size was arrived at	4
Quantitative variables	11	Explain how quantitative variables were handled in the analyses. If applicable, describe which groupings were chosen and why	5-6
Statistical methods	12	(*a*) Describe all statistical methods, including those used to control for confounding	5-7
		(*b*) Describe any methods used to examine subgroups and interactions	N/A
		(*c*) Explain how missing data were addressed	N/A
		(*d*) If applicable, explain how matching of cases and controls was addressed	4
		(*e*) Describe any sensitivity analyses	N/A
**Results**			
Participants	13	(a) Report numbers of individuals at each stage of study—*e.g.* numbers potentially eligible, examined for eligibility, confirmed eligible, included in the study, completing follow-up, and analysed	7
		(b) Give reasons for non-participation at each stage	7
		(c) Consider the use of a flow diagram	Fig.1

Descriptive data	14	(a) Give characteristics of study participants (*e.g.* demographic, clinical, social) and information on exposures and potential confounders	7 Tab.1
		(b) Indicate the number of participants with missing data for each variable of interest	N/A
Outcome data	15	Report numbers in each exposure category, or summary measures of exposure	Tab.1
Main results	16	(*a*) Give unadjusted estimates and, if applicable, confounder-adjusted estimates and their precision (*e.g.*, 95% confidence interval). Make clear which confounders were adjusted for and why they were included	8-10 Tab.2, S1,S2,S3
		(*b*) Report category boundaries when continuous variables were categorized	N/A
		(*c*) If relevant, consider translating estimates of relative risk into absolute risk for a meaningful time period	N/A
Other analyses	17	Report other analyses done—*e.g.* analyses of subgroups and interactions, and sensitivity analyses	N/A
**Discussion**			
Key results	18	Summarise key results with reference to study objectives	10-11
Limitations	19	Discuss limitations of the study, taking into account sources of potential bias or imprecision. Discuss both direction and magnitude of any potential bias	11
Interpretation	20	Give a cautious overall interpretation of results considering objectives, limitations, multiplicity of analyses, results from similar studies, and other relevant evidence	11-14
Generalisability	21	Discuss the generalisability (external validity) of the study results	14
**Other information**			
Funding	22	Give the source of funding and the role of the funders for the present study and, if applicable, for the original study on which the present article is based	14

SUPPLEMENTARY FIGURES

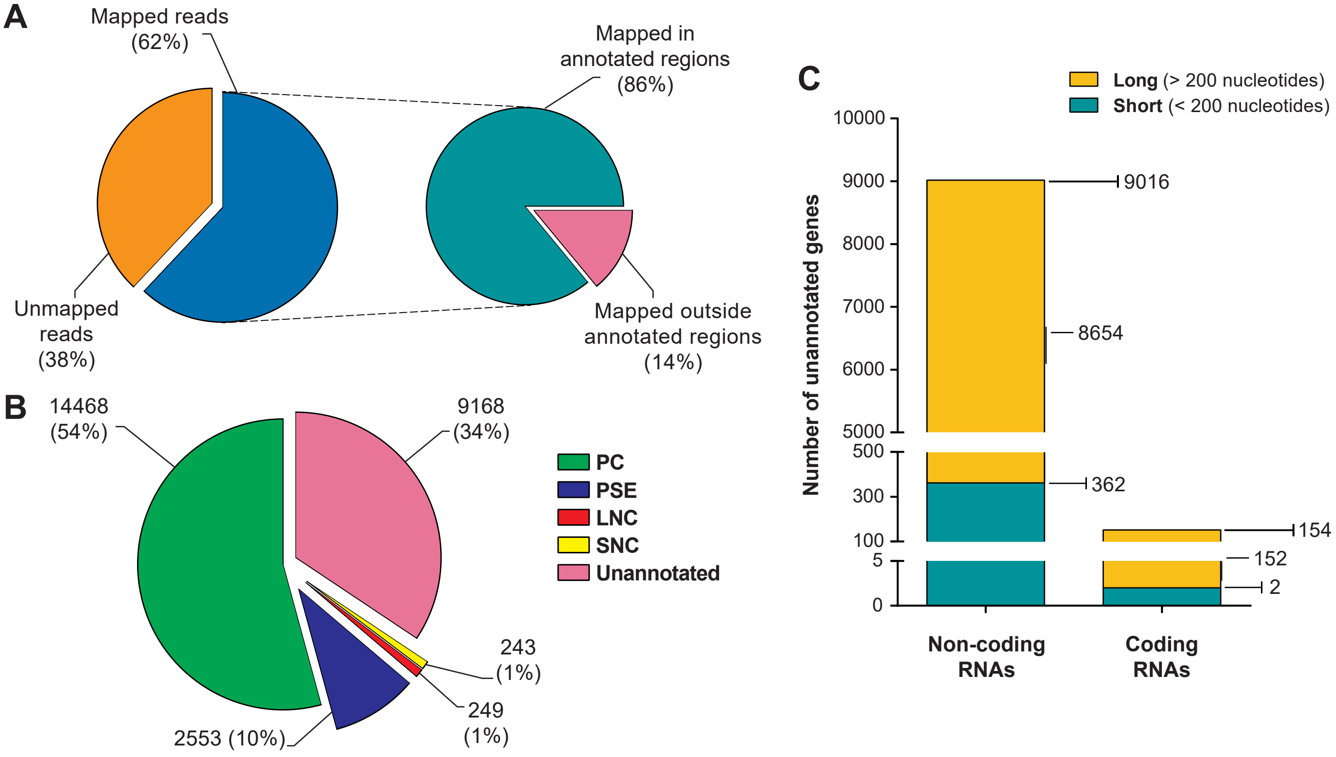

Figure S1 | Read and gene feature statistics. A. For each sample (STEMI *n* = 15, NSTEMI *n* = 15), on average, 62% of sequenced reads were aligned; 14% of them mapped to genomic sequences where the presence of exonic regions has not been defined yet. **B**. Expressed genes were grouped in biotypes based on Ensembl annotation: PC, Protein Coding genes; PSE, Pseudogenes; LNC, Long Non-Coding genes; SNC, Short Non-Coding genes; and Unannotated, putative novel intergenic transcripts. **C**. The coding potential of the unannotated transcripts was assessed and 154 were found to be putative novel coding genes, being the remaining non-coding RNAs.

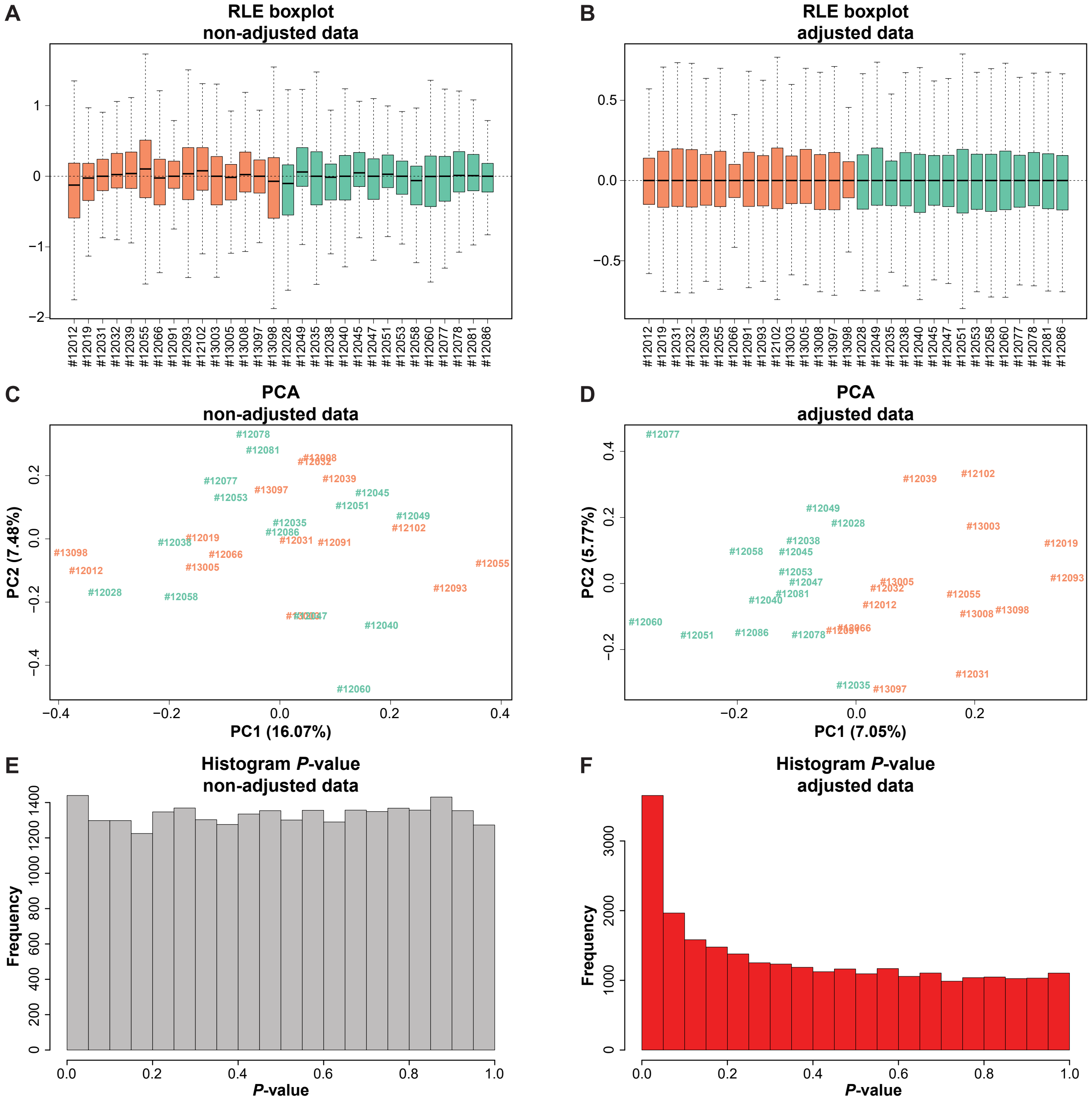

Figure S2 | Quality control assessment after adjustment for unwanted confounding variables. Panels **A** and **B** display, respectively, the relative log expression (RLE) boxplots for non-adjusted and adjusted expression data: the median (black dash) centred to zero for adjusted data indicates proper normalization. Panels **C** and **D** show the scatter plots and the percentage of variance explained by the first two principal components (PC) of the principal component analysis (PCA) performed on non-adjusted and adjusted expression data: PCA on adjusted data shows a clear separation between STEMI (n = 15) and NSTEMI (n = 15) patients. Panels **E** and **F** display the distribution of the nominal *P*-values for testing differential expression between STEMI *vs.* NSTEMI for the non-adjusted and adjusted models. The overall uniform distribution of the *P*-values in the non-adjusted model suggests that confounding factors are likely affecting differential expression analysis. Conversely, the adjusted model presents the expected uniform distribution for the bulk of non-differentially expressed genes and a spike near zero, which corresponds to those differentially expressed. In panels **A**–**D**, orange and green boxes or labels refer, respectively, to STEMI and NSTEMI subjects.

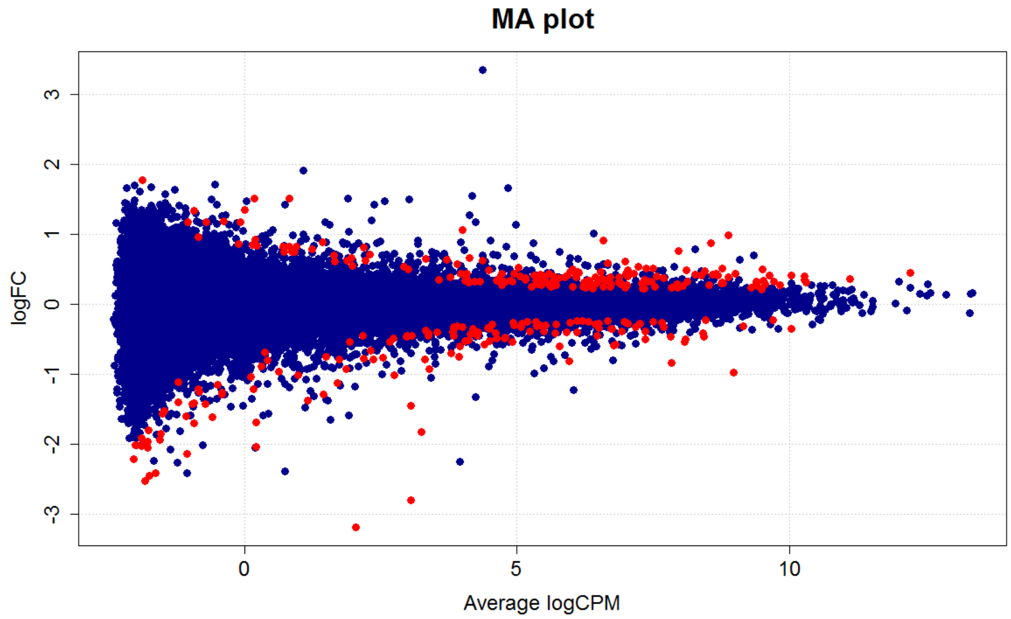

Figure S3 | MA-Plot. The plot shows the relationship between the log_2 fold change (logFC) of STEMI (n = 15) *vs.* NSTEMI (n = 15) with the average expression of each gene across samples [measured as the log_2 count per million (CPM) of reads]; significant calls (red dots) span from low to high expression.

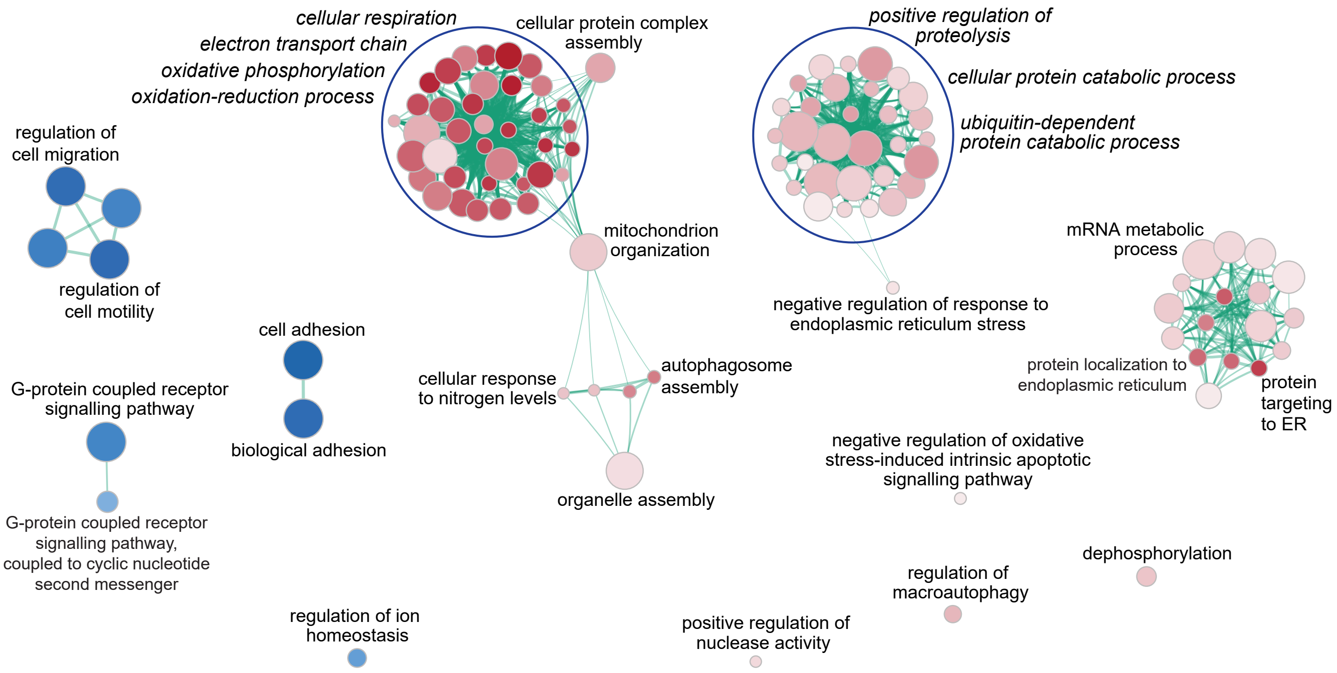

Figure S4 | Enrichment map of the gene-sets stemming from the analysis on the dataset not corrected for admission cardiac troponin I (cTnI). A functional enrichment investigation on genome-wide transcript profiles was done by Gene Set Enrichment Analysis (GSEA). We used as gene ranking metric the likelihood ratio statistics of the differential expression analysis performed by negative binomial Generalized Linear Model (GLM), in STEMI (n = 15) *vs.* NSTEMI (n = 15) patients, matched for age, sex, and cardiovascular risk factors, without correction for cTnI level on admission. To visually interpreting biological data from GSEA analysis, a network of the most significant Gene Ontology Biological Process (GO-BP) terms (adjusted $P < 0.05$) was drawn. The node colour associates to STEMI (red) or NSTEMI (blue) phenotype; node gradient colour is proportional to node significance, from lower (light) to higher (dark); node size is proportional to the gene-set size. Edge thickness is proportional to the similarity between two gene-sets, for a cut-off of 0.25 of the combined Jaccard plus Overlap coefficient.

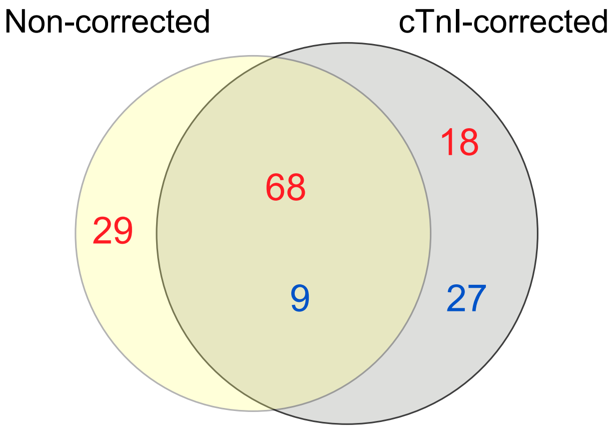

Figure S5 | Scaled Venn diagram showing relations between the non-corrected and the cTnI-corrected GSEA datasets. Gene-sets specifically enriched in the cTnI-corrected dataset were 18 for STEMI (in red) and 27 for NSTEMI (blue), respectively (right part of the grey circle; *Fig. 4* in the main text shows the corresponding network analysis). Overlapping GO-BP terms, *i.e.*, enriched both in the non-corrected and in the cTnI-corrected datasets, were 68 for STEMI and 9 for NSTEMI (intersection between the 2 circles; see *Fig. S6* below for network visualization). The 29 gene-sets unique to the non-corrected analysis (left part of the yellow circle; see *Fig. S7* below for network visualization) were associated only with STEMI.

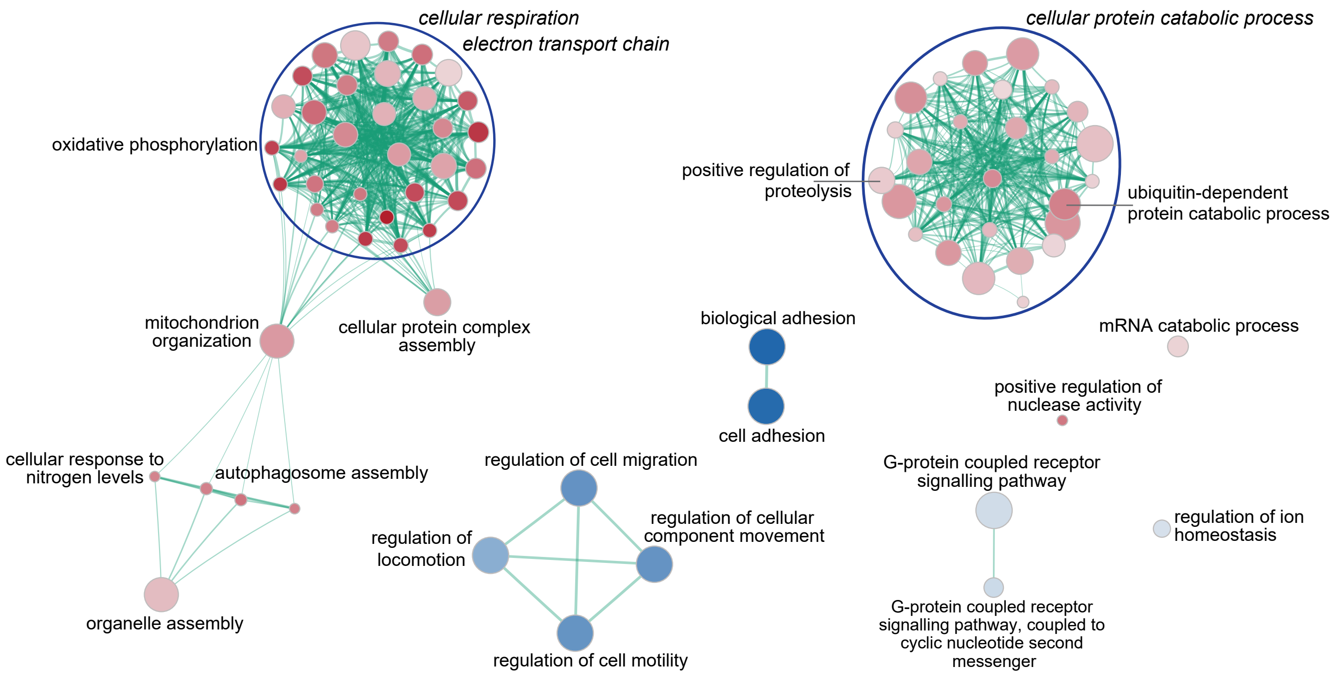

Figure S6 | Enrichment map of gene-sets that overlapped in the GSEA results obtained from the non-corrected and the cTnI-corrected datasets. Functional enrichments on genome-wide profiles were made by GSEA, using as gene ranking metric the likelihood ratio statistics of the differential expression analyses performed by negative binomial GLM, either correcting or not for cTnI level on admission in STEMI (n = 15) *vs.* NSTEMI (n = 15) patients, matched for age, sex, and cardiovascular risk factors. Overlapping results between the enrichments are depicted here. Colour codes are the same as in *Fig. S4*.

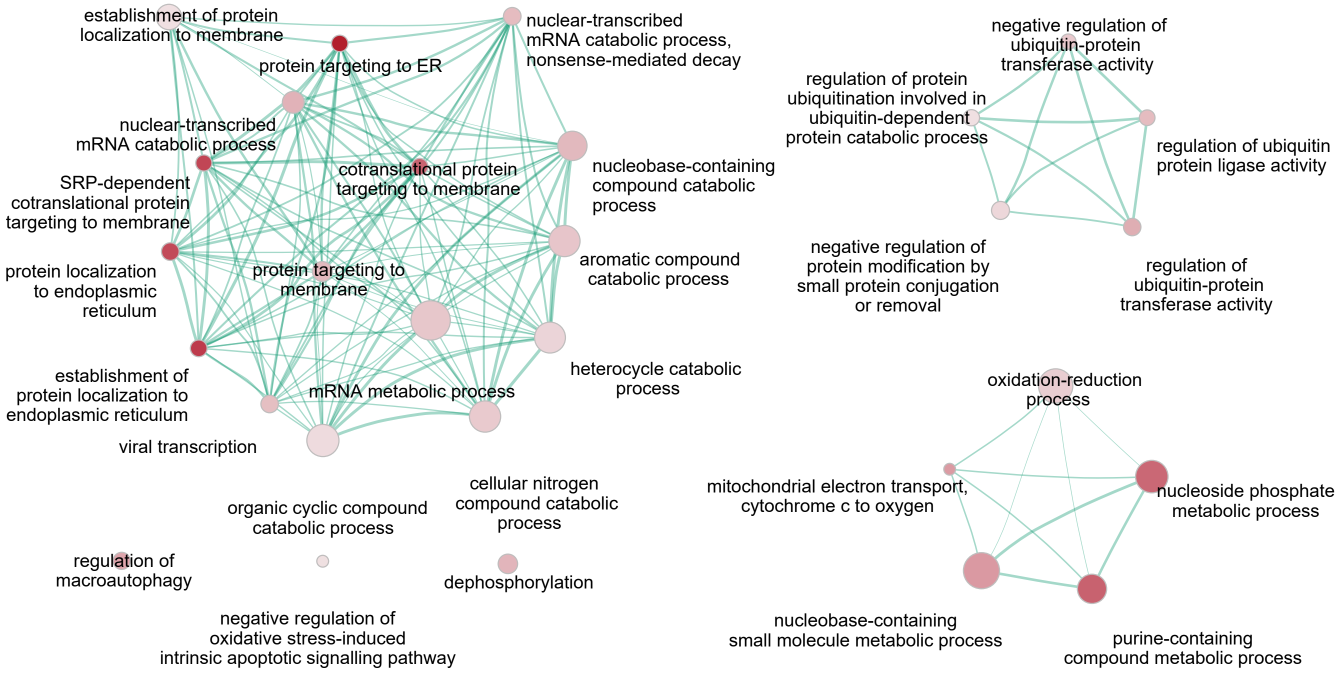

Figure S7 | Enrichment map of gene-sets that were present only in the GSEA dataset not corrected for cTnI. Functional enrichments on genome-wide profiles were made by GSEA, using as gene ranking metric the likelihood ratio statistics of the differential expression analyses performed by negative binomial GLM, either correcting or not for cTnI level on admission, in STEMI (n = 15) *vs.* NSTEMI (n = 15) patients, matched for age, sex, and cardiovascular risk factors. Results unique to the enrichment derived from the uncorrected analysis are depicted here: these include gene-sets overrepresented in STEMI patients only. Colour codes are the same as in *Fig. S4*.

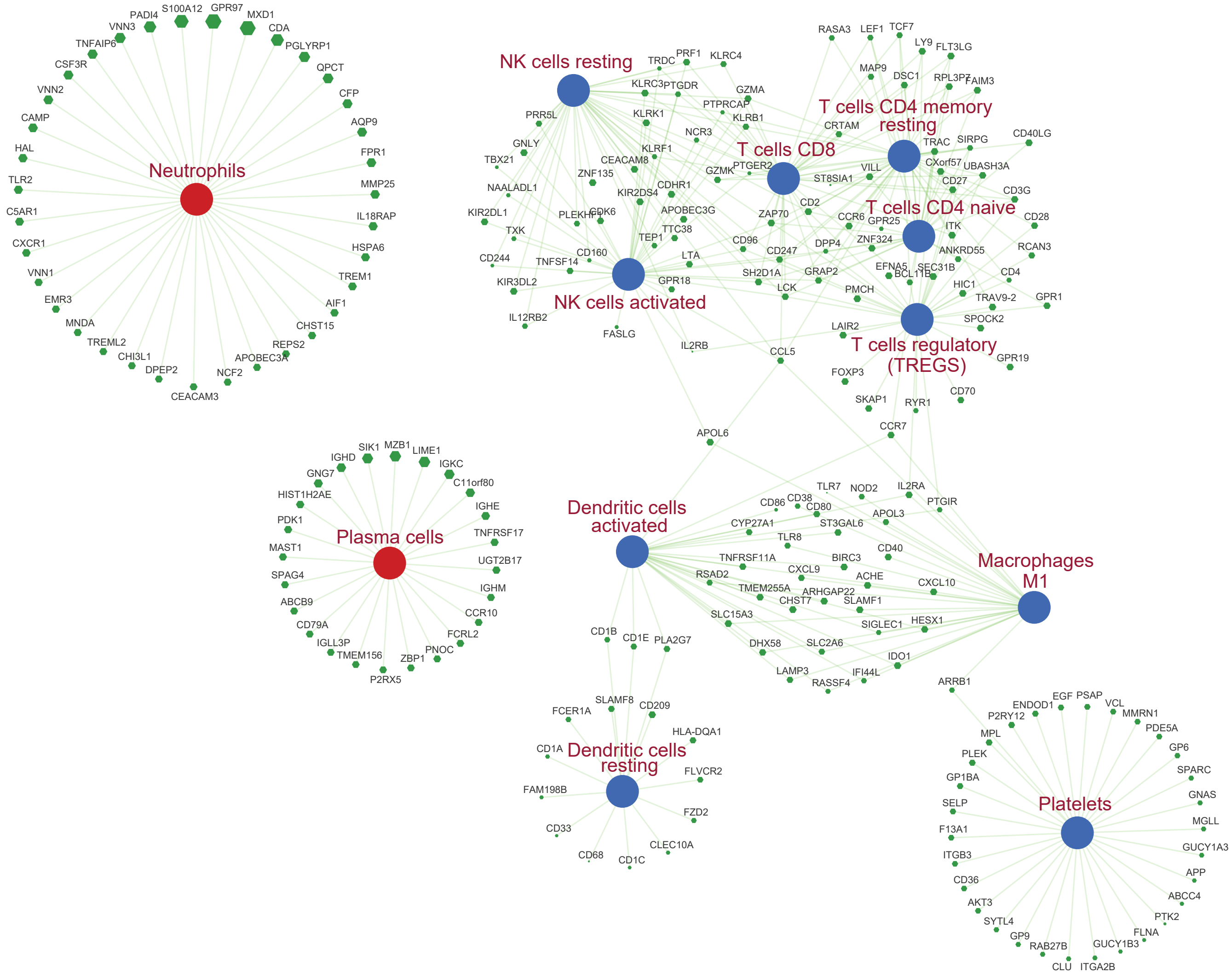

Figure S8 | Cell-type enrichment map. Functional enrichment on genome-wide transcript profiles was made by GSEA, using as gene ranking metric the likelihood ratio statistics of the differential expression analysis performed by negative binomial GLM, in STEMI (*n* = 15) *vs.* NSTEMI (*n* = 15) patients matched for age, sex, and cardiovascular risk factors, before correction for cTnI levels on admission. Results from cell-type enrichment analysis were drawn as a network. Cell-type node and label size are proportional to the combined score (from higher to lower). Rounded nodes represent over-expressed genes in STEMI (red) and NSTEMI (blue) associated with specific cell-types.

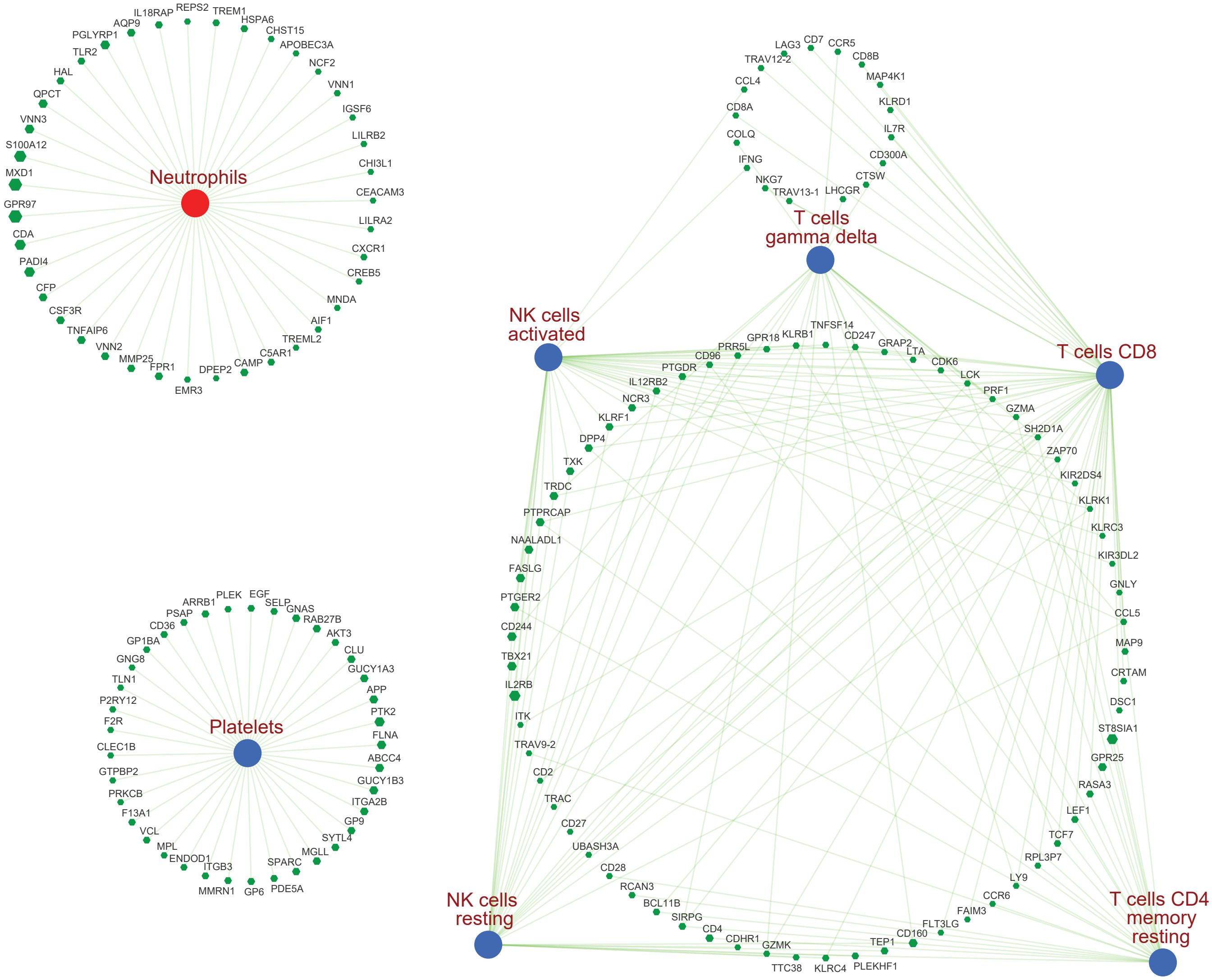

Figure S9 | Cell-type enrichment map after correction for admission cTnI levels. Functional enrichment on genome-wide profiles was made by GSEA, using as gene ranking metric the likelihood ratio statistics of the differential expression analysis performed by negative binomial GLM, in STEMI (n = 15) *vs.* NSTEMI (n = 15) patients matched for age, sex, and cardiovascular risk factors, after correction for cTnI levels on admission. See *Fig. S8* for details.

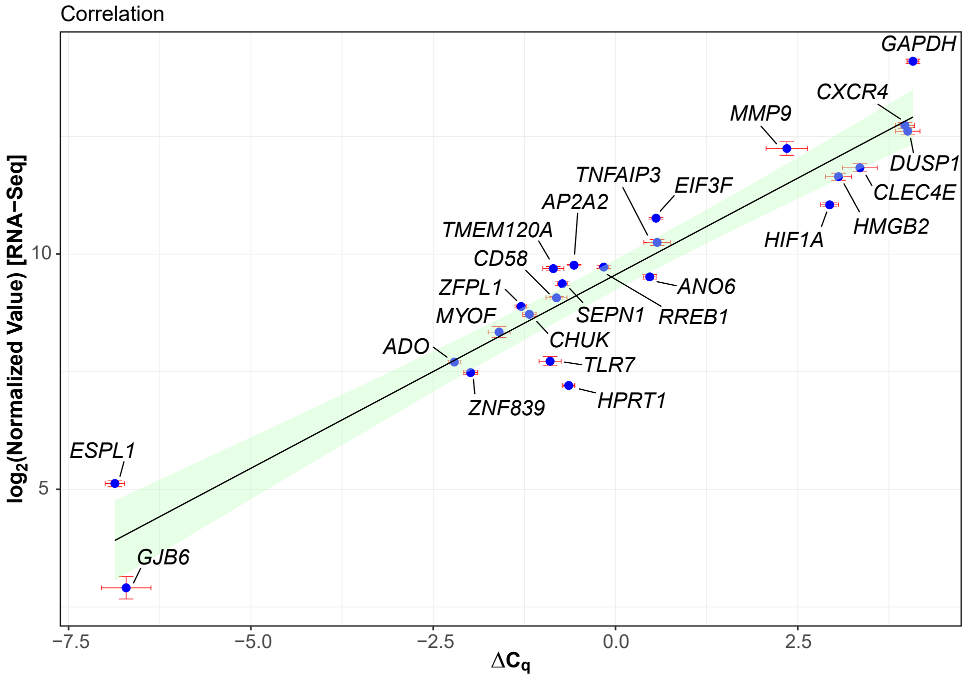

Figure S10 | Technical validation of RNA-Seq data by real-time RT-qPCR assays. We performed a confirmatory analysis of gene expression data, selecting genes that spanned from low to high-abundance expression levels, endogenous control genes, differentially expressed genes and genes associated with the cTnI peak in STEMI (n = 15) *vs.* NSTEMI (n = 15) patients matched for age, sex, and cardiovascular risk factors. The relative expression of 24 genes was evaluated in patients from the study cohort using RT-qPCR Single Assays. On the x-axis is the ΔCq (using the mean expression of *AP2A2* and *EIF3F* as reference gene); on the y-axis is the normalized counts on RNA-Seq. We computed the Pearson's correlation coefficients (r) and reported within the plot along with the significance P-value. Blue dots are the averaged expression values, while red bars are the standard errors; the 95% confidence interval of the trend line is represented by the green area.

Supplementary Tables

Table S1 | Whole-genome differential gene expression analysis in peripheral blood from STEMI *vs.* NSTEMI patients. The table shows annotations and statistics for all the genes detected by RNA-Seq. Differential gene expression was assessed both correcting or not for cTnI levels on admission. Significant comparisons for an FDR-adjusted *P*-value < 0.05 are highlighted in green. Genes are sorted by statistical significance in the non-corrected model. For each gene, the table reports Ensembl and HGNC unique identifiers, official gene symbol, and name, Ensembl biotype, $\log_2$ fold-difference ($\log_2$FC STEMI *vs.* NSTEMI), the average expression level in Counts Per Million mapped reads (CPM), likelihood ratio (LR) statistics, nominal and FDR-adjusted significance *P*-values. *See Supplementary Dataset 1.xlsx online.*

Table S2 | Gene-set enrichment analysis. (*a*) GSEA on GO-BP terms on uncorrected and cTnI-corrected statistic gene ranks. **(*b*)** Cell-type enrichment analysis on uncorrected and cTnI-corrected statistic gene ranks. Significant tests for an FDR-adjusted *P*-value < 0.05 are highlighted in green. Gene sets are sorted by statistical significance in the non-corrected model. For each gene set, the table reports its functional annotation term (GO-BP or blood cell-type), GO identifier, list of genes belonging to it, size, enrichment scores (raw and normalized), significance *P*-values (nominal, FDR- and FWER-adjusted), and position in the ranked list. *See Supplementary Dataset 2.xlsx.*

Table S3 | Regression analysis of gene expression levels on admission for cTnI peak prediction. Significant tests for an FDR-adjusted *P*-value < 0.05 are highlighted in green. Gene sets are sorted by statistical significance in the non-corrected models. For each gene, the table reports the coefficients of determination R^2 unadjusted or adjusted for the number of predictors, the regression β coefficient, and significance *P*-values (nominal and FDR-adjusted). *See Supplementary Dataset 3.xlsx online.*

Supplementary References

1 Langmead, B., Trapnell, C., Pop, M. & Salzberg, S. L. Ultrafast and memory-efficient alignment of short DNA sequences to the human genome. *Genome Biol*. **10**, R25 (2009).
2 Kim, D. *et al.* TopHat2: accurate alignment of transcriptomes in the presence of insertions, deletions and gene fusions. *Genome Biol*. **14**, R36 (2013).
3 Flicek, P. *et al.* Ensembl 2014. *Nucleic Acids Res*. **42**, D749-755 (2014).
4 Roberts, A., Pimentel, H., Trapnell, C. & Pachter, L. Identification of novel transcripts in annotated genomes using RNA-Seq. *Bioinformatics*. **27**, 2325-2329 (2011).
5 Trapnell, C. *et al.* Differential gene and transcript expression analysis of RNA-seq experiments with TopHat and Cufflinks. *Nat Protoc*. **7**, 562-578 (2012).
6 Andersen, C. L., Jensen, J. L. & Orntoft, T. F. Normalization of real-time quantitative reverse transcription-PCR data: a model-based variance estimation approach to identify genes suited for normalization, applied to bladder and colon cancer data sets. *Cancer Res*. **64**, 5245-5250 (2004).
